# Isolation of a phosphinidene sulfide and selenide†

**DOI:** 10.1039/d5sc04352b

**Published:** 2025-07-10

**Authors:** Chenyang Hu, Maren Pink, Jose M. Goicoechea

**Affiliations:** a Department of Chemistry, Indiana University 800 East Kirkwood Ave. Bloomington Indiana 47405 USA jgoicoec@iu.edu; b Department of Chemistry, University of Oxford, Chemistry Research Laboratory 12 Mansfield Rd. Oxford OX1 3TA UK

## Abstract

We describe the synthesis of an isolable phosphinidene sulfide and phosphinidene selenide (BnArNP=Ch; Ch = S, Se) by reaction of a sterically protected phosphinidene oxide (BnArNP=O) with Lawesson's or Woollins' reagents, respectively. These compounds reveal short P=E bonds consistent with a high degree of p_π_–p_π_ double bond character. The pendant amido group on the phosphorus atom plays an important role in their stabilization through nitrogen lone pair donation into the P–Ch π* orbital, and evidence for isomerism about the N–P bond was observed for both species. Dimeric intermediates featuring mixed phosphorus(iii)/phosphorus(v) centers could be isolated and structurally authenticated during the course of these studies providing some insight into the mechanism of formation of the titular compounds.

## Abstract 2

We describe the synthesis of an isolable phosphinidene sulfide and phosphinidene selenide (BnArNP=Ch; Ch = S, Se) by reaction of a sterically protected phosphinidene oxide (BnArNP=O) with Lawesson's or Woollins' reagents, respectively.

## Graphical abstract

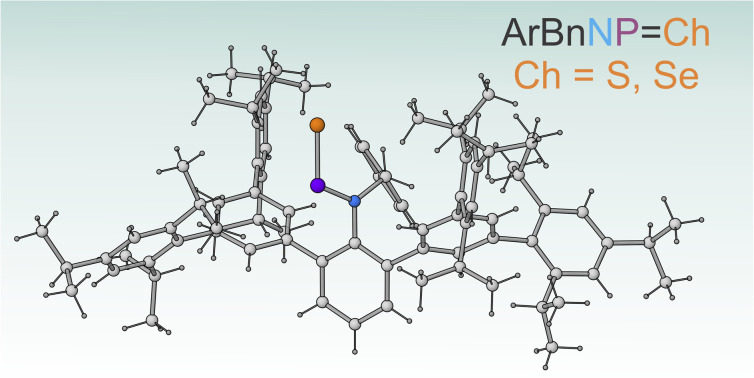

## Introduction

**Fig. 1:**
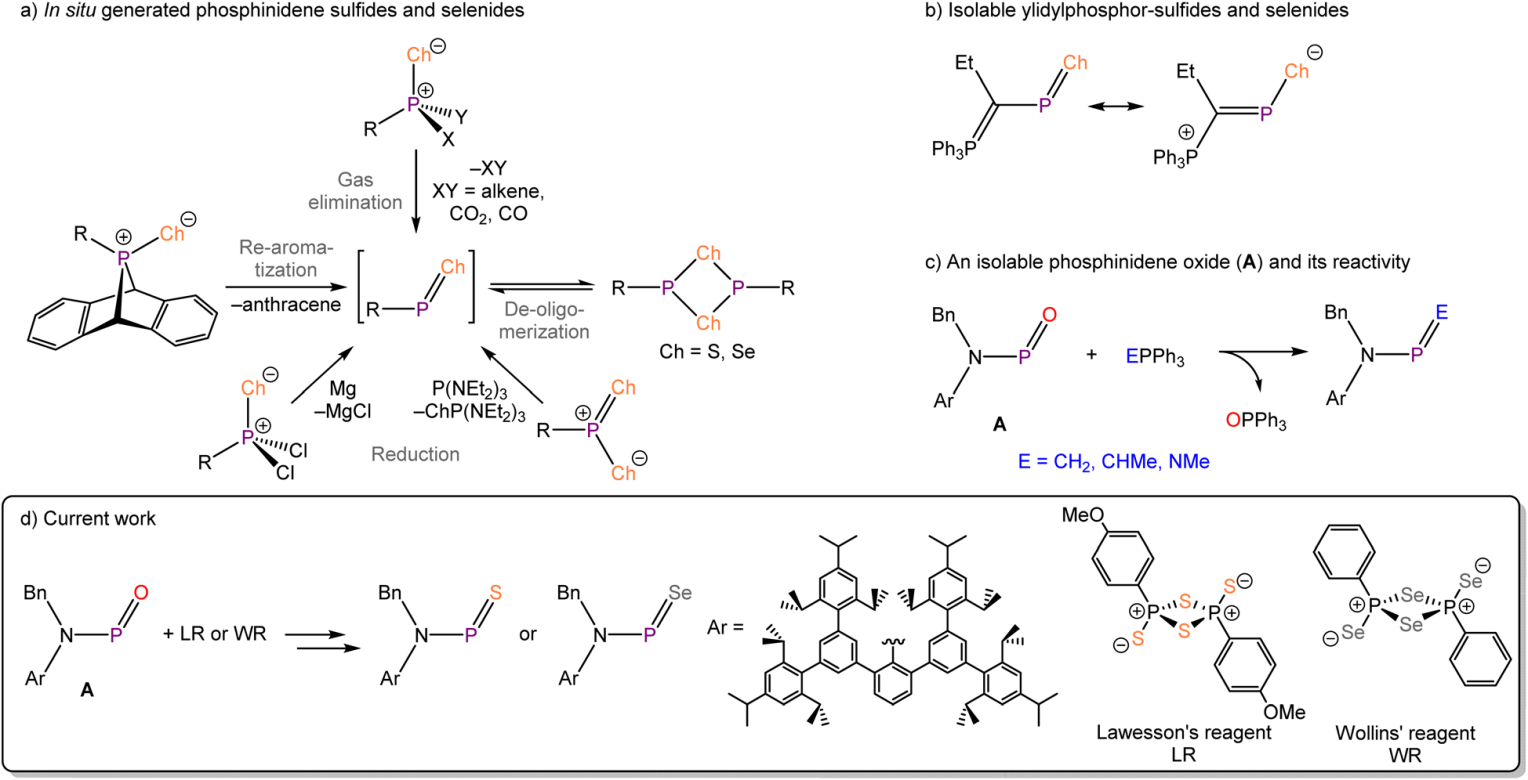
(a) Reported methods for the generation of phosphinidene sulfides and selenides; (b) an isolable phosphinidene sulfide and selenide supported by an ylidyl substituent; (c) a phosphinidene oxide and its phospha–Wittig reactivity; (d) current work: isolation of a phosphinidene sulfide and selenide.

Phosphinidenes (R–P) are regarded as heavier analogues of carbenes (R_2_C) due to the diagonal relationship between phosphorus and carbon.^1–13^ Their oxidized phosphorus(iii) counterparts, phosphinidene chalcogenides (R–P=Ch; where Ch = group 16 element), are also known to exhibit carbene-like reactivity.^14,15^ That being said, the chemical properties of phosphinidene chalcogenides remain poorly understood on account of their inherent instability under ambient conditions. The polarity of phosphorus–chalcogen bonds, and their weak π-bonding character, makes them highly reactive, which often leads to deleterious dimerization or oligomerization reactions. Given the difficulties in isolating heavier phosphinidene chalcogenides, they have thus far only been observed at extremely low temperatures (3–15 K) in inert gas matrices (Fig. 1a).^16^

Two different approaches towards the chemical stabilization of phosphinidene chalcogenides have been previously explored in the chemical literature. The first of these, which we term the intermolecular approach, involves the trapping of *in situ* generated phosphinidene chalcogenides with chemical reagents (*e.g.* dienes).^14,17–27^ Using this approach, the formation of a transient phosphinidene chalcogenide can be inferred by the resulting products (often a heterocycle of some kind), however this does not allow for the isolation of the reactive P=Ch bond. The second strategy involves the intramolecular electronic stabilization of the P=Ch moiety by adjacent metal centres,^28–31^ or electron-donating R substituents.^32–35^ This latter strategy was successfully employed by Schmidpeter and co-workers to access the only structurally authenticated R–P=S and R–P=Se compounds reported to date (Fig. 1b). Both compounds are stabilized by electron-delocalization of the ylidyl substituent into the P=Ch π* orbital and were found to be moderately stable in solution for short periods of time.^34,35^

Recently, we reported the first example of an isolable phosphinidene oxide using a bulky amido supporting ligand for the stabilization of the highly reactive P(iii)=O bond (BnArNP=O).^36^ During these studies, it became apparent that the nitrogen atom lone pair plays an important role in the stabilization of the reactive P=O moiety. We have since gone on to show that this compound can act as an electrophilic phosphinidene precursor for the synthesis of phosphorus-main group element double bonds (Fig. 1c).^37^ This motivated us to explore the feasibility of employing the same ligand to support heavier phosphinidene chalcogenides. Herein, we expand this strategy and report the transformation of this phosphinidene oxide to its sulfide and selenide analogues using Lawesson's and Woollins' reagents, respectively (Fig. 1d). It is worth noting that recently Tan described the isolation of heavier antimony-containing analogues, the stibinidene chalcogenides (M^s^Fluid*Sb=Ch; Ch = S–Te; M^s^Fluid* is a bulky hydrindacene substituent) in which the reactive Sb=Ch bonds are protected by an extremely bulky hydrindacene ligand.^38^

## Results and discussion

Inspired by the phospha–Wittig reaction, we initiated our studies by reacting phosphinidene oxide, A, with either triphenylphosphine sulfide (SPPh_3_) or trimethylphosphine sulfide (SPMe_3_). Given the similar electronic structures of SPR_3_ and phosphorus ylides, we reasoned that these reagents would allow chalcogen atom transfer to the phosphorus atom. To our surprise, unlike phosphorus ylides, which react with A instantly,^37^ no reaction was observed between SPPh_3_ or SPMe_3_ and A, even at elevated temperatures. This suggests that phosphine sulfides lack the requisite nucleophilicity to undergo reactions with phosphinidene oxides. Similarly, no Wittig-type reactions between phosphine sulfides and carbonyl compounds have been reported to date, further supporting the inertness of phosphine sulfides in such transformations.

Given the structural and electronic similarity between A and carbonyl compounds, we trialled other strategies which have been shown to be effective in the chalcogenation of carbonyl-containing species. Lawesson's reagent (LR) is well established as a versatile thionation reagent. The mechanism which gives rise to its effective reactivity, involves the dissociation of the dimeric structure and the *in situ* generation of a dithioxo-phosphorane.^39^ We envisioned that the use of Lawesson's reagent could facilitate the conversion of our phosphinidene oxide, A, to its sulfide analogue, mirroring the thionation reactivity observed towards carbonyl compounds.

**Scheme 1:**
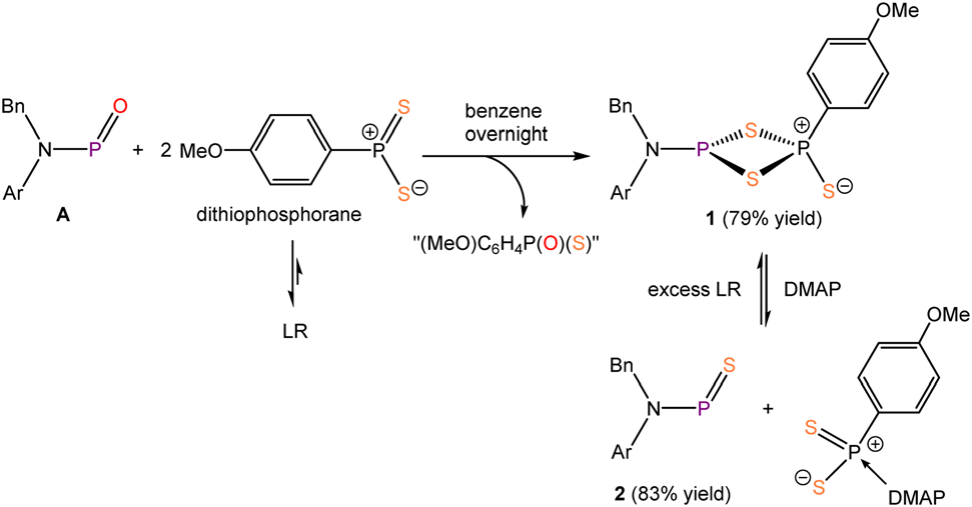
Synthesis of compounds 1 and 2.

Reaction of 0.5 equivalents of LR with 1 equivalent of compound A (Scheme 1) gave rise to multiple products, as evidenced by *in situ*^31^P{^1^H} NMR spectroscopy. Addition of excess LR drove conversion to a major product which features a set of doublets at 42.5 and 47.8 ppm with a ^2^*J*_P–P_ coupling constant of 93.2 Hz. The proton-coupled ^31^P NMR spectrum revealed that the resonance at 42.5 ppm splits into a doublet of triplets, with a ^3^*J*_P–H_ coupling constant of 15.2 Hz, indicating that it originates from the LR fragment (*i.e.* coupling to a *p*-methoxy-phenyl group is observed). These data suggests that this product, 1, is a dimeric phosphinidene sulfide, incorporating a dithio-phosphorane unit derived from LR dissociation. Similar dimeric structures have been reported previously for germanium and tin, formed *via* the reaction of germylenes, stannylenes or stannylene sulfides with LR.^40^ The reaction also affords an intractable mixture of side-products which we believe to be oligomers of (MeO)C_6_H_4_P(O)(S) and (MeO)C_6_H_4_P(S)_2_, as previously reported in the literature.^41^

The structure of compound 1 was confirmed by single crystal X-ray diffraction.† As expected, the structure revealed a four-membered P_2_S_2_ ring containing one P(iii) centre and one P(v) centre. Given the poor quality of the crystal and the presence of extensive positional disorder, the discussion of bond metric data is not viable, however the connectivity is consistent with a dimeric compound (see ESI, Fig. S27†). It is worth noting that a related dimeric compound ^Dipp^TerP(μ-S)_2_P(S)^Dipp^Ter, was recently reported by Hering-Junghans and co-workers (^Dipp^Ter 2,6-(2,6-_*i*_Pr_2_–C_6_H_3_)_2_–C_6_H_3_).^42^

During the purification of 1, a minor product caught our attention. Upon removal of the excess LR from the reaction mixture by washing with MeCN, two new singlet resonances appeared at 477.0 and 472.1 ppm in the ^31^P{^1^H} NMR spectrum in a 4.28 : 1 ratio, the chemical shifts of these resonances comparable favourably with the R–P=S compound reported by Schmidpeter.^34^ These two peaks are also similar to those observed for the phosphinidene oxide A, which also displays two ^31^P{^1^H} NMR singlet resonances arising from *cis*–*trans* isomers. Based on this spectral similarity, we assigned these peaks to phosphinidene sulfide 2. A dynamic equilibrium exists between compounds 1, 2 and LR, with the equilibrium favouring the formation of compound 1. Notably, while the removal of excess LR shifts the equilibrium toward compound 2, washing with MeCN alone was insufficient to eliminate all the residual LR.

We hypothesized that introducing an additional reagent capable of consuming LR while leaving compound 2 intact would provide the additional thermodynamic driving force needed to generate 2. LR and its analogues are known to react with Lewis bases, forming four-coordinate monomeric adducts.^43,44^ For this purpose, 4-dimethylaminopyridine (DMAP) was selected as an external Lewis base to neutralize LR. Satisfactorily, upon the addition of excess DMAP to compound 1, *in situ*^31^P{^1^H} NMR spectroscopy revealed the complete consumption of 1, and increased signal intensity for compound 2, together with a new singlet at 109.4 ppm. Independent reaction of LR with DMAP in THF confirmed that the new ^31^P{^1^H} resonance corresponds to the monomeric LR-DMAP adduct. These findings provide strong support for the success of our synthetic strategy. Subsequently, a one-pot synthesis of compound 2 was performed by sequential addition of LR and DMAP to compound A, affording 2 in excellent yields (83%) on a 50 mg scale (Scheme 1). Addition of excess LR to 2 leads to the reformation of 1 again, further confirming the equilibrium nature of this transformation.

**Fig. 2:**
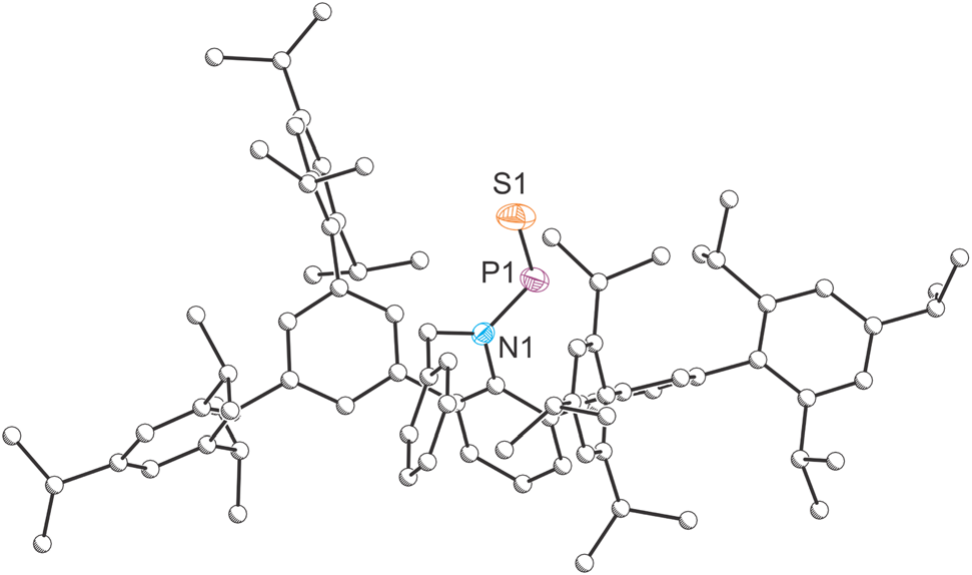
Single-crystal X-ray structures of 2. Carbon atoms depicted with an arbitrary radius. Thermal ellipsoids pictured at 50% probability level. Positional disorder and hydrogen atoms are removed for clarity.

The solid-state molecular structure of compound 2 (Fig. 2) was determined by single crystal X-ray diffraction. The crystal structure reveals a *trans*-arrangement of the BnArN–P=S moiety about the N–P bond, consistent with the solid-state structure of A. The P=S bond length in 2, 1.9166(14) Å, is significantly shorter than the P–S bond in Schmidpeter's ylide-substituted phosphinidene sulfide (1.981(2) Å), which the authors attribute to a significant contribution from the “dipolar resonance formula”.^34^ Base-stabilized phosphinidene sulfides, such as an intramolecularly stabilized species recently reported by Nikonov, also exhibit longer P–S bonds (*e.g.* 1.998(2) Å).^45^ Notably, the P=S bond in 2 is also shorter than the sum of double bond covalent radii (1.96 Å)^46^ and the P(v)–S bond in SPPh_3_ (1.9554 Å).^47^ It is worth noting that the short P–S bond in SPPh_3_ arises from hyperconjugation of the sulphur atom lone pairs into a doubly degenerate set of P–C σ* antibonding orbitals and that, consequently, such bonds cannot be referred to as double bonds. In contrast, in compound 2, the P=S bond length is attributed to a strong π-interaction between phosphorus and sulphur, reinforcing the P=S double bond character (*vide infra*). Notably, the nitrogen atom still retains its planar geometry 
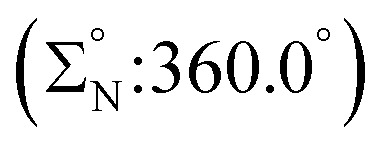
, consistent with A and previously reported amido phosphaalkenes and phosphaimines.^48–50^ The N–P bond length (1.674(3) Å) falls between that of single and double bond, closely resembling previously reported N–P bond length in amido phosphaalkenes and phosphaimines. These structural features indicate that the nitrogen atom lone pair is delocalized into P–S π* orbital, which aligns with the restricted N–P bond rotation observed in the aforementioned NMR studies.

**Fig. 3:**
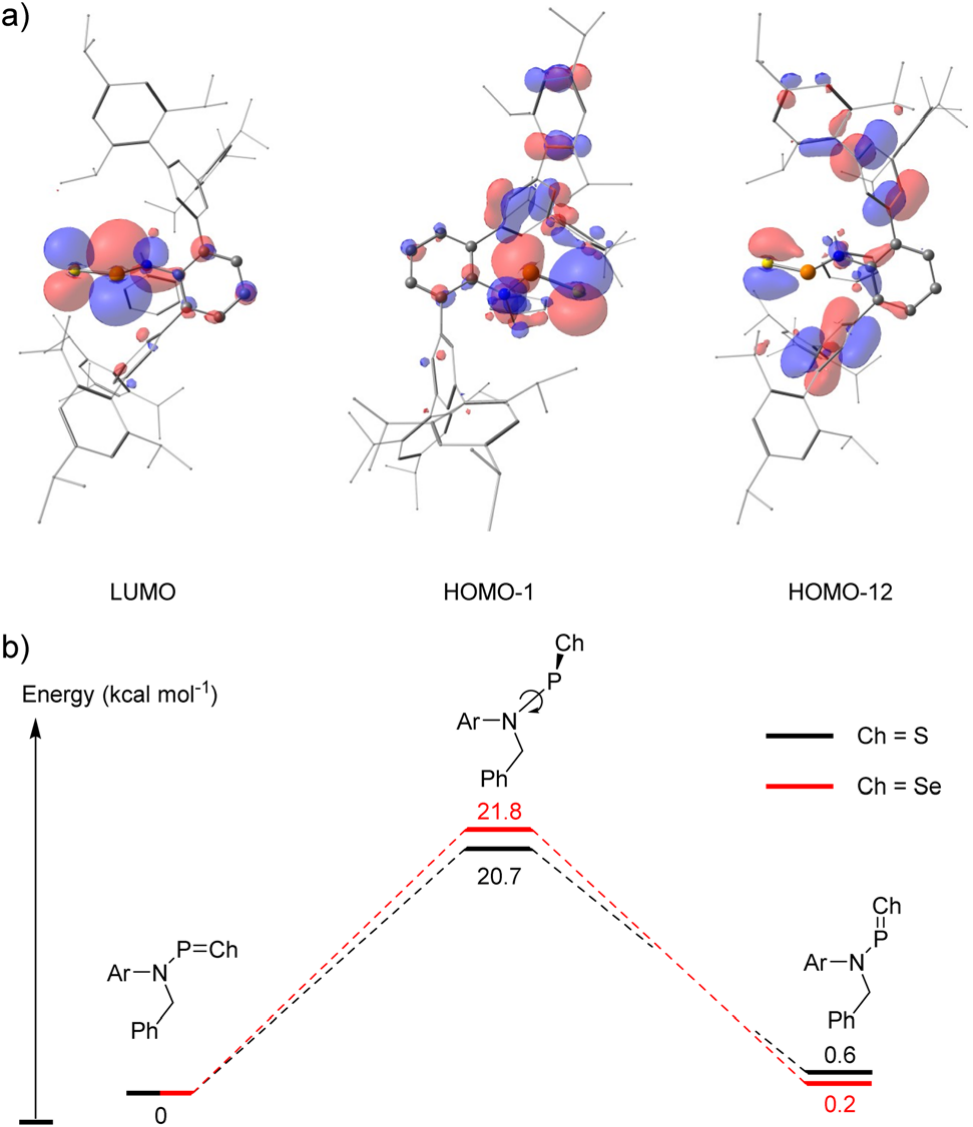
(a) Selected Kohn–Sham molecular orbital depictions (isovalue = 0.03) for compound 2; (b) calculated N–P bond rotation energy profiles for 2 and 4.

To gain deeper insight into the electronic structure of 2, density functional theory (DFT) calculations were performed at the M06-2X/Def2-SVP level of theory. The optimized geometry is in good agreement with that obtained by X-ray crystallography (*e.g.* P–S: 1.941 Å; N–P: 1.689 Å; 
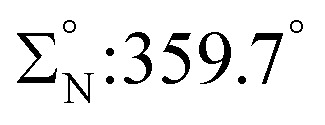
). While the LUMO orbital reveals a highly localized P–S π* orbital, the P–S π orbital is diffuse and low in energy, observed as the HOMO−12. Additionally, one of the lone pairs on sulphur is distinctly characterized in the HOMO−1 (Fig. 3a). Natural bond orbital (NBO) analysis further confirmed the presence of a well-defined σ-bond and π-bond within the P=S moiety (Fig. S29 in ESI†). The π-interaction is predominantly composed of p-orbital overlap between phosphorus (96.97% p-character) and sulphur (98.14% p-character), with a significant polarization toward sulphur (70.47%). Second-order perturbation theory analysis revealed a back-donation energy of 36.05 kcal mol^−1^ from the nitrogen atom lone pair to the P–S π* orbital, which is comparable to that of A (41.12 kcal mol^−1^). The back-donation was also evidenced by the low occupancy of nitrogen lone pair (1.699 e^−^) and the partially filled P–S π* orbital (0.229 e^−^). Mayer bond indices corroborate these findings, indicating a bond order of 1.09 for N–P and 1.81 for P=S. Since the electronic structure of 2 closely resembles that of A, a similar energy barrier for N–P bond rotation should also be expected. Energy calculations at SMD-M06-2X/def2-TZVP//M06-2X/def2-SVP level revealed a rotation barrier of 20.7 kcal mol^−1^, closely matching that of A (21.8 kcal mol^−1^). The *trans*-isomer of 2 is thermodynamically favoured by 0.6 kcal mol^−1^, agreeing with the crystallographic observed *trans*-geometry for the N–P bond (Fig. 3b).

The thermodynamic profile for the equilibrium between 1, 2 and LR was further investigated using DFT calculations (see Scheme S8 in ESI† for full details). Reaction of 2 with 0.5 equivalents of LR to generate compound 1 was found to be spontaneous and thermodynamically favoured by 2.9 kcal mol^−1^, which aligns well with experimental observations, as compound 1 is the predominant species in the reaction mixture. DMAP-induced LR cleavage is determined to be an exothermic reaction (−6.5 kcal mol^−1^). By coupling these reactions, the overall transformation (formation of 2 from reaction of 1 and DMAP) favours the formation of 2, with an overall free energy change of −3.6 kcal mol^−1^. These computational results are in strong agreement with experimental observations, where the addition of DMAP effectively shifts the equilibrium between compounds 1, 2 and LR, to afford 2.

**Scheme 2:**
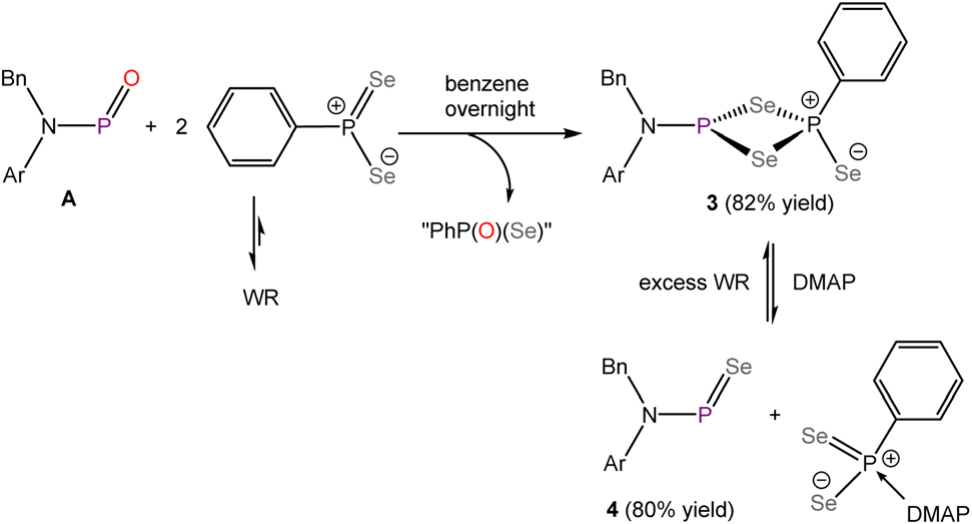
Synthesis of compounds 3 and 4.

The successful thionation of A prompted us to explore the use of Woollins' Reagent (WR) to access a phosphinidene selenide. Woollins' reagent is the selenium analogue of Lawesson's reagent and is widely utilized in organic synthesis for the selenation of carbonyl compounds.^51^ Building on insights gained from the reaction between compound A and LR, we treated A with excess WR in benzene (Scheme 2). After stirring overnight, a bright-yellow solution was obtained, and *in situ*^31^P NMR spectroscopy revealed a doublet at 46.0 ppm with a ^2^*J*_P–P_ coupling constant of 80.6 Hz, and a doublet of triplets at −46.0 ppm (^2^*J*_P–P_ = 80.6 Hz and ^3^*J*_P–H_ = 17.0 Hz). The similarity of this ^31^P NMR spectrum to that of compound 1 suggests the formation of the dimeric structure 3. Since Woollins' reagent can also be cleaved using a Lewis base,^52^ we envisioned that addition of DMAP can promote the formation of phosphinidene selenide 4, following an analogous mechanism to that proposed for the formation of 2. Indeed, upon the addition of excess DMAP to the reaction mixture (Scheme 2), the solution rapidly changed colour to light red. *In situ*^31^P{^1^H} NMR spectroscopy revealed two singlet resonances at 535.4 and 528.6 ppm in a 4.81 : 1 ratio, closely resembling the ^31^P{^1^H} NMR spectrum of compounds A and 2. Extended ^31^P{^1^H} NMR scans further revealed the presence of selenium satellites, with a ^31^P–^77^Se coupling constant of 788.1 Hz, confirming the formation of phosphinidene selenide 4. While no signals were observed in the ^77^Se NMR spectrum at room temperature, a broad doublet appeared at 1081.6 ppm when the NMR spectrum was recorded at −45 °C. These observations are tentatively attributed to the chemical exchange process between the *cis*–*trans* isomers at room temperature, leading to broad and weak signals. Upon cooling, the chemical exchange process is effectively frozen, resulting in enhanced spectral resolution and signal intensity in the ^77^Se NMR spectrum. Upon re-addition of excess Woollins' reagent, 4 can cleanly convert back to 3.

**Fig. 4:**
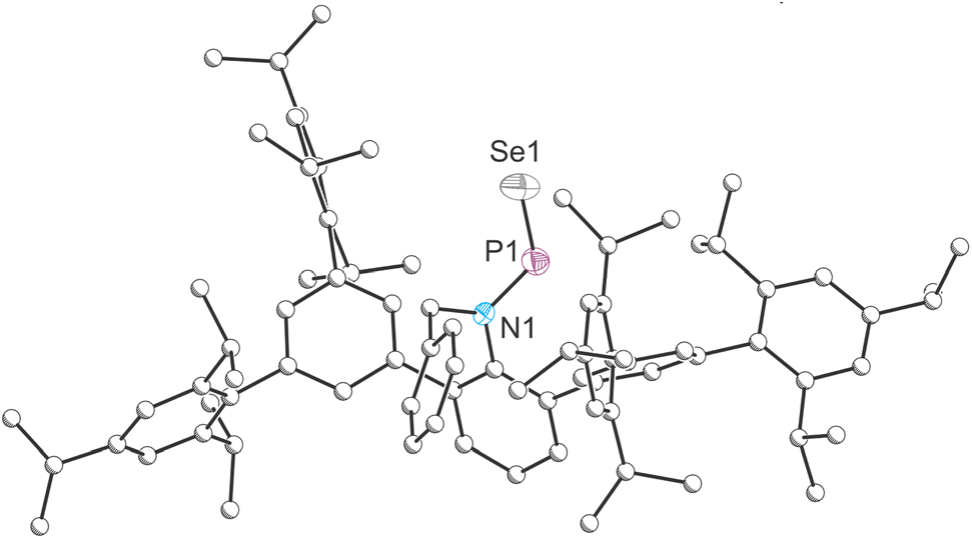
Single-crystal X-ray structure of 4. Carbon atoms depicted with an arbitrary radius. Thermal ellipsoids pictured at 50% probability level. Positional disorder and hydrogen atoms are removed for clarity.

The structures of 3 and 4 were authenticated by single crystal X-ray diffraction and are comparable to those of 1 and 2, demonstrating structural consistency across the series (Fig. 4). As with its sulphur-containing analogue, phosphinidene selenide 4 adopts a *trans*-geometry in solid state. The P=Se bond length in 4 was determined to be 2.0662(15) Å, which again is notably shorter that the ylidyl-functionalised analogue (2.129(2) Å). This bond is also shorter than the phosphorus(v)–Se bond (2.106(1) Å) in SePPh_3_,^53^ due to presence of a strong P–Se π interaction. The planar geometry of the nitrogen atom 
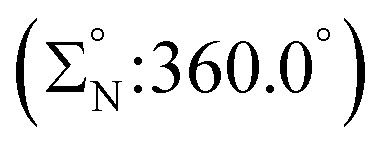
 and the short N–P bond length (1.669(4) Å) are consistent with those observed for A and 2, indicating the back-donation from the nitrogen lone pair to P–Se σ* antibonding orbital.

**Fig. 5:**
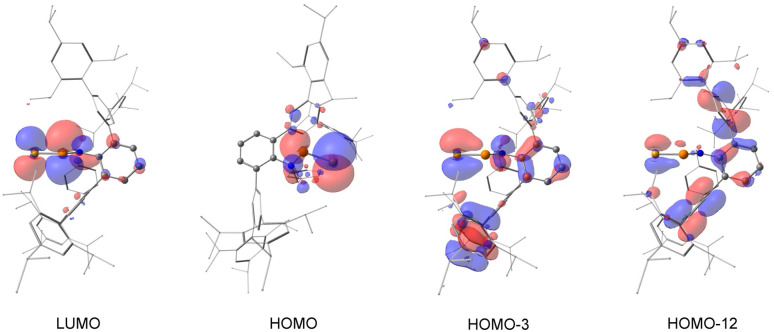
Selected Kohn–Sham molecular orbital depictions (isovalue = 0.03) for compound 4.

DFT calculations were conducted to elucidate the electronic structure of 4. While the LUMO of 4 is predominantly composed of the P–Se π* interaction, the HOMO represents the lone pair of the selenium atom (Fig. 5). The P–Se π bond is highly delocalized and is observed in the HOMO−3 and HOMO−12. Natural bond orbital (NBO) analysis confirmed the presence of both σ- and π-bonding orbital between phosphorus and selenium (Fig. S29 in ESI†). Due to the similar electronegativity of Se (2.55) and S (2.58), the p-type π orbital remains polarized, with Se contributing 64.72% and P contributing 35.28%, which is comparable to the values determined for 2.

Second-Order Perturbation Theory analysis revealed a back-donation energy of 19.04 kcal mol^−1^ for nitrogen lone pair donating to the P–Se π* bond and 12.49 kcal mol^−1^ to σ* bond, in agreement with the crystal structure of 4. Notably, while the back-donation to π* bond is weaker than calculated for 2 (36.05 kcal mol^−1^), the σ* back-donation is much stronger in 4 (12.49 kcal mol^−1^*versus* 3.52 kcal mol^−1^ in 2). We attribute this difference to the lower electronegativity and larger atomic orbital size of Se. The P–Se π-bond in 4 is less polar, which disfavours the π* back-donation, whereas the orbital mismatch between phosphorus and selenium weakens the σ-bond, thereby enhancing the σ* back-donation. Energy calculations determined a *cis*–*trans* isomerization barrier of 21.8 kcal mol^−1^, with the *trans*-isomer being energetically favoured by 0.2 kcal mol^−1^, consistent with the crystallographic structure of 4. While the back-donation in 4 is weaker than that in 2, it may seem counterintuitive that the rotation barrier is higher in 4 (21.8 kcal mol^−1^*versus* 20.7 kcal mol^−1^ in 2). This can be rationalized by the longer P–Se bond length in 4 (2.0662(15) Å) compared to the P–S bond in 2 (1.9166(14) Å), which introduces additional steric hindrance in the transition state. This energy barrier also matches the observed ^31^P{^1^H} NMR spectrum of 4, which is similar to those observed for A and 2. Mayer bond indices suggest a bond order of 1.11 for the N–P bond and 1.75 for the P=Se bond, reinforcing the proposed bonding model.

## Conclusions

Following the successful isolation of a free phosphinidene oxide, we herein report the isolation of its heavier analogues, a phosphinidene sulfide 2 and a phosphinidene selenide 4, further expanding the chemistry of phosphorus(iii)–chalcogen double bonds. Lawesson's reagent (LR) and Woollins' reagent (WR) are widely employed in organic synthesis for the transformation of carbonyl compounds into corresponding thio- and seleno-carbonyl derivatives. Given the similarity between carbonyl compounds and phosphinidene oxide A, we have shown that these reagents are also effective in converting phosphinidene oxide A to the corresponding sulfide and selenide. Upon reaction of phosphinidene oxide A with excess LR or WR, the *in situ* generated phosphinidene chalcogenide overreacted with LR or WR, forming dimeric structures 1 and 3, which features a P_2_Ch_2_ four-membered ring motif. Addition of excess DMAP to 1 or 3 introduces an external thermodynamic driving force, facilitating the generation of monomeric phosphinidene sulfide 2 and selenide 4. Crystal structures of both 2 and 4 revealed a *trans*-geometry within the ArBnN–P=Ch moiety and back-donation from the nitrogen atom lone pair to P–Ch π* orbital, which is also observed in A. DFT calculations further support the presence of significant back-donation, and an energetically significant rotation barrier was determined for 2 (20.7 kcal mol^−1^) and 4 (21.8 kcal mol^−1^), which is comparable to that observed in A.

## Experimental section

### General synthetic methods

All reactions and product manipulations were carried out under an inert atmosphere of argon or dinitrogen using standard Schlenk-line or glovebox techniques (MBraun UNIlab glovebox maintained at <0.1 ppm H_2_O and <0.1 ppm O_2_). Benzene (anhydrous, Sigma-Aldrich), toluene (Fisher Chemical, HPLC grade), hexane (Fisher Chemical, HPLC grade) and acetonitrile (Fisher Chemical, HPLC grade) were purified using a Pure Process Technology (PPT) solvent purification system (SPS). C_6_D_6_ (Aldrich, 99.5%) and d_8_-toluene (Aldrich, 99%) were distilled over sodium/benzophenone. CDCl_3_ was distilled over CaH_2_. All dry solvents were stored under argon in gas-tight ampoules over activated 3 Å molecular sieves. Compound A was synthesized following the procedure described in the literature.^36^ Lawesson's reagent, Woollins' reagent and DMAP were purchased from TCI and Sigma-Aldrich, respectively, and used as received.

### Analytical techniques

NMR spectra were acquired on a Bruker 500 MHz Avance Neo, a Varian 500 MHz Inova, or a Varian 400 MHz Inova NMR spectrometer. Chemical shifts (*δ*) are reported in parts per million (ppm). ^1^H and ^13^C NMR spectra are referenced to TMS using residual protio-solvent resonance (^1^H NMR C_6_D_6_: *δ* = 7.16 ppm, ^13^C NMR C_6_D_6_: *δ* = 128.06 ppm; ^1^H NMR CDCl_3_: *δ* = 7.26 ppm, ^13^C NMR CDCl_3_: *δ* = 77.16 ppm, ^1^H NMR d_8_-toluene: *δ* = 2.08 ppm). ^31^P NMR spectra were externally referenced to an 85% solution of H_3_PO_4_ in H_2_O. ^77^Se NMR spectra were externally referenced to a solution of SeMe_2_ in C_6_D_6_. High-resolution mass spectra were recorded on a Thermo Q-Exactive Plus (ESI-TOF, positive ion mode) instrument at the Mass Spectrometry Facility of the Department of Chemistry of Indiana University.

### Synthesis of 1

To a solid mixture of A (30 mg, 0.025 mmol, 1.00 eq.) and Lawesson's reagent (20 mg, 0.049 mmol, 2 eq.), benzene (0.5 mL) was added. The mixture was stirred overnight at room temperature and monitored by ^31^P{^1^H} NMR spectroscopy. Once compound A was fully consumed, the mixture was filtered, and MeCN (20 mL) was added to the filtrate. The mixture was left in a −35 °C freezer overnight to crystalize. The supernatant was removed, and the resulting solid was dried *in vacuo* to afford 1 (28 mg, 0.020 mmol; 79% yield) as a colourless powder. Single crystals of 1 suitable for X-ray diffraction were obtained by slow evaporation of a concentrated hexane solution. ^1^H NMR (500 MHz, C_6_D_6_): *δ* (ppm) 1.12 (d, ^3^*J*_H–H_ = 6.8 Hz, 12H; CH(C*H*_3_)_2_), 1.15 (d, ^3^*J*_H–H_ = 6.8 Hz, 18H; CH(C*H*_3_)_2_), 1.24–1.29 (m, 42H; CH(C*H*_3_)_2_), 2.85 (sept, ^3^*J*_H–H_ = 6.8 Hz, 4H; C*H*(CH_3_)_2_), 3.04–3.10 (m, 8H; C*H*(CH_3_)_2_), 3.12 (s, 3H; OC*H*_3_), 5.44 (s, 2H; NC*H*_2_Ph), 6.59–6.62 (m, 2H; ArH), 6.68–6.75 (m, 3H; ArH), 7.00 (t, ^3^*J*_H–H_ = 7.8 Hz, 1H; ArH), 7.16 (s, overlapping with C_6_D_6_, 8H, ArH), 7.17 (d, ^4^*J*_H–H_ = 1.7 Hz, 2H; ArH), 7.19 (d, ^4^*J*_P–H_ = 1.7 Hz, 2H; ArH), 7.22 (d, ^4^*J*_H–H_ = 1.7 Hz, 2H; ArH), 7.30 (d, ^3^*J*_H–H_ = 7.8 Hz, 2H; ArH), 7.56 (d, ^3^J_H–H_ = 8.6 Hz, 2H; ArH), 8.26 (dd, ^3^*J*_H–P_ = 15.2 Hz, ^3^*J*_H–H_ = 8.6 Hz, 2H; ArH). (Accurate integration was not possible as, in solution, 1 is in equilibrium with 2 and LR). ^13^C{^1^H} NMR (125 MHz, C_6_D_6_): *δ* (ppm) 24.40, 24.43, 24.68, 25.04, 25.23, (s; CH(*C*H_3_)_2_) 30.71, 30.99, 31.11, 34.84, 34.93 (s; *C*H(CH_3_)_2_), 52.80 (br; N*C*H_2_Ph) 54.88 (s; O*C*H_3_), 113.81 (d, ^1^*J*_P–C_ = 16.7 Hz), 120.89, 128.59, 129.63, 131.53, 131.72, 132.39, 133.49, 133.54, 133.61, 135.20 (d, ^1^*J*_P–C_ = 17.1 Hz), 136.39, 137.07, 137.38, 140.28, 141.39, 141.90, 142.39 (d, ^2^*J*_P–C_ = 6.6 Hz), 147.00, 148.53, 163.07 (s; ArC). ^31^P NMR (162 MHz, C_6_D_6_): *δ* (ppm) 42.5 (dt, ^2^*J*_P–P_ = 93.2 Hz ^3^*J*_P–H_ = 15.2 Hz; PS*P*(S)C_6_H_4_OMe), 47.8 (d, ^3^*J*_P–P_ = 93.2 Hz; *P*SP(S)C_6_H_4_OMe). ^31^P{^1^H} NMR (162 MHz, C_6_D_6_): *δ* (ppm) 42.48 (d, ^3^*J*_P–P_ = 93.2 Hz; PS*P*(S)C_6_H_4_OMe), 47.84 (d, ^3^*J*_P–P_ = 93.2 Hz; *P*SP(S)C_6_H_4_OMe). HRMS (*m*/*z*): [M + H]^+^ calcd. For C_92_H_116_NOP_2_S_3_, 1408.7689; found 1408.7670.

### Synthesis of 2

To a solid mixture of A (50 mg, 0.042 mmol, 1.00 eq.) and Lawesson's reagent (40 mg, 0.099 mmol, 2.3 eq.), benzene (0.7 mL) was added. The mixture was stirred overnight at room temperature and monitored by 31P{1H} NMR spectroscopy. Once compound A was fully consumed, the mixture was filtered, and DMAP (20 mg, 0.164 mmol, 3.9 eq.) was added to the filtrate. After stirring the mixture for 30 min at room temperature, the formation of a precipitate was observed. The mixture was filtered again, and MeCN (20 mL) was added to the filtrate. The mixture was left in a −35 °C freezer overnight to crystalize. The supernatant was removed, and the resulting solid was dried *in vacuo* to afford 2 (42 mg, 0.035 mmol; 83% yield) as a light-green powder. Single crystals of 2 suitable for X-ray diffraction were obtained by slow evaporation of a concentrated hexane solution. ^1^H NMR (500 MHz, C_6_D_6_): *δ* (ppm) 1.16 (d, ^3^*J*_H–H_ = 6.8 Hz, 12H; CH(C*H*_3_)_2_), 1.20–1.22 (m, 24H; CH(C*H*_3_)_2_), 1.28–1.32 (m, 36H; CH(C*H*_3_)_2_), 2.87 (sept, ^3^*J*_H–H_ = 6.8 Hz, 4H; C*H*(CH_3_)_2_), 2.93–3.06 (m, 8H; C*H*(CH_3_)_2_), 5.46 (s, 2H; NC*H*_2_Ph), 6.61 (t, ^3^*J*_H–H_ = 7.6 Hz, 2H; ArH), 6.74 (t, ^3^*J*_H–H_ = 7.6 Hz, 2H; ArH), 6.79–6.83 (m, 1H; ArH), 6.95 (t, ^3^*J*_H–H_ = 7.8 Hz, 1H; ArH), 7.10 (t, ^4^*J*_H–H_ = 1.6 Hz, 2H; ArH), 7.16–7.17 (m, overlapping with C_6_D_6_, 8H; ArH), 7.19 (t, ^4^*J*_H–H_ = 1.6 Hz, 2H; ArH), 7.21 (t, ^4^*J*_H–H_ = 1.6 Hz, 2H; ArH), 7.31 (t, ^3^*J*_H–H_ = 7.8 Hz, 2H; ArH). (Accurate integration was not possible due to the presence of the minor isomer, 2-*cis*). ^13^C{^1^H} NMR (125 MHz, C_6_D_6_): *δ* (ppm) 24.17, 24.37, 24.41, 24.45, 24.70, 24.81, 25.02, 25.09 (s; CH(*C*H_3_)_2_), 30.86, 30.89, 30.99, 31.00, 31.11, 34.88 (s; *C*H(CH_3_)_2_), 51.02 (br; N*C*H_2_Ph), 120.72, 120.78, 128.59, 129.37, 130.53, 131.19, 131.37, 131.81, 131.91, 132.10, 135.78, 137.06, 137.24, 139.86, 140.61 (d, *J*_P–C_ = 5.2 Hz), 140.94, 141.57, 146.78, 146.90, 148.60 (s; ArC). ^31^P{^1^H} NMR (202 MHz, C_6_D_6_): *δ* (ppm) 477.0 (s; 2-*trans*), 472.1 (s; 2-*cis*). Ratio 2-*trans* : 2-*cis* = 4.28 : 1. HRMS (*m*/*z*): [M + H]^+^ calcd. For C_85_H_109_NPS, 1206.8013; found 1206.8017.

### Synthesis of 3

To a solid mixture of A (30 mg, 0.025 mmol, 1.00 eq.) and Woollins' reagent (26 mg, 0.049 mmol, 2 eq.), benzene (0.5 mL) was added. The mixture was stirred overnight at room temperature and monitored by ^31^P{^1^H} NMR spectroscopy. Once compound A was fully consumed, the mixture was filtered, and MeCN (20 mL) was added to the filtrate. The mixture was left in a −35 °C freezer overnight to crystalize. The supernatant was removed, and the resulting solid was dried *in vacuo* to afford 3 (31 mg, 0.020 mmol; 82% yield) as a bright-yellow powder. Single crystals of 3 suitable for X-ray diffraction were obtained by slow evaporation of a concentrated hexane solution. ^1^H NMR (500 MHz, C_6_D_6_): *δ* (ppm) 1.11 (d, ^3^*J*_H–H_ = 6.9 Hz, 18H; CH(C*H*_3_)_2_), 1.17 (d, ^3^*J*_H–H_ = 6.9 Hz, 18H; CH(C*H*_3_)_2_), 1.24–1.30 (m, 36H; CH(C*H*_3_)_2_), 2.85 (sept, ^3^*J*_H–H_ = 6.9 Hz, 4H; C*H*(CH_3_)_2_), 3.01–3.14 (m, 8H; CH̲(CH_3_)_2_), 5.53 (s, 2H; NCH̲_2_Ph), 6.69–6.75 (m, 3H; ArH), 6.97–7.02 (m, 3H; ArH), 7.16 (s, overlapping with C_6_D_6_, 8H; ArH), 7.18 (d, ^4^*J*_H–H_ = 1.7 Hz, 2H; ArH), 7.20 (d, ^4^*J*_H–H_ = 1.7 Hz, 2H; ArH), 7.25 (t, ^4^*J*_H–H_ = 1.7 Hz, 2H; ArH), 7.29 (d, ^3^*J*_H–H_ = 7.7 Hz, 2H; ArH), 7.53–7.58 (m, 3H; ArH), 8.42 (dd, ^3^*J*_H–P_ = 17.0 Hz, ^3^*J*_H–H_ = 7.2 Hz, 2H; ArH). (Accurate integration was not possible as, in solution, 3 is in equilibrium with 4 and WR). ^13^C{^1^H} NMR (125 MHz, C_6_D_6_): *δ* (ppm) 24.40, 24.44, 24.68, 25.04, 25.32 (s; CH(*C*H_3_)_2_), 30.77, 30.84, 30.90, 31.05, 31.11, 34.84, 34.88 (s; *C*H(CH_3_)_2_), 55.24 (br; N*C*H_2_Ph), 120.71, 120.82, 120.96, 128.59, 129.63, 131.46, 131.56, 131.62, 131.82, 132.39, 136.26, 136.95, 137.05, 137.17, 137.38, 138.87 (d, ^1^*J*_C–P_ = 25.3 Hz), 140.13, 140.88, 141.40, 141.54, 141.90, 142.40 (d, ^2^*J*_C–P_ = 6.2 Hz) 143.84 (br), 146.79, 146.91, 146.96, 147.05, 147.10, 148.56, 148.63 (s; ArC). ^31^P NMR (202 MHz, C_6_D_6_): *δ* (ppm) −46.0 (dt, ^2^*J*_P–P_ = 80.6 Hz, ^3^*J*_P–H_ = 17.0 Hz; PSe*P*(Se)Ph), 46.0 (d, ^2^*J*_P–P_ = 80.6 Hz, *P*SeP(Se)Ph). ^31^P{^1^H} NMR (202 MHz, C_6_D_6_): *δ* (ppm) −45.95 (d, ^2^*J*_P–P_ = 80.6 Hz; PSe*P*(Se)Ph), 46.04 (d, ^2^*J*_P–P_ = 80.6 Hz; *P*SeP(Se)Ph). HRMS (*m*/*z*): [M + H]^+^ calcd. For C_91_H_114_NP_2_Se_3_, 1520.5925; found 1520.5954.

### Synthesis of 4

To a solid mixture of A (50 mg, 0.042 mmol, 1.00 eq.) and Woollin's reagent (40 mg, 0.075 mmol, 1.8 eq.), benzene (0.7 mL) was added. The mixture was stirred overnight at room temperature and monitored by ^31^P{^1^H} NMR spectroscopy. Once compound A was fully consumed, the mixture was filtered, and DMAP (20 mg, 0.164 mmol, 3.9 eq.) was added to the filtrate. After stirring the mixture for 30 min at room temperature, the formation of a precipitate was observed. The mixture was filtered again, and MeCN (20 mL) was added to the filtrate. The mixture was left in a −35 °C freezer overnight to crystalize. The supernatant was removed, and the resulting solid was dried *in vacuo* to afford 4 (42 mg, 0.033 mmol; 80% yield) as a light-red powder. Single crystals of 4 suitable for X-ray diffraction were obtained by slow evaporation of a concentrated hexane solution at room temperature. ^1^H NMR (500 MHz, C_6_D_6_): *δ* (ppm) 1.15 (d, ^3^*J*_H–H_ = 6.8 Hz, 12H; CH(C*H*_3_)_2_), 1.18 (d, ^3^*J*_H–H_ = 6.8 Hz, 12H; CH(C*H*_3_)_2_), 1.21 (d, ^3^*J*_H–H_ = 6.8 Hz, 12H; CH(C*H*_3_)_2_), 1.28 (d, ^3^*J*_H–H_ = 6.8 Hz, 24H; CH(C*H*_3_)_2_), 1.30–1.32 (m, 12H; CH(C*H*_3_)_2_), 2.86 (sept, ^3^*J*_H–H_ = 6.8 Hz, 4H; C*H*(CH_3_)_2_), 2.92–3.07 (m, 8H; C*H*(CH_3_)_2_), 5.49 (s, 2H; NC*H*_2_Ph), 6.61 (t, ^3^*J*_H–H_ = 7.5 Hz, 2H; ArH), 6.72–6.75 (m, 2H; ArH), 6.79–6.81 (m, 1H; ArH), 6.94 (t, ^3^*J*_H–H_ = 7.7 Hz, 1H; ArH), 7.09 (t, ^4^*J*_H–H_ = 1.5 Hz, 2H; ArH), 7.16–7.17 (m, overlapping with C_6_D_6_, 8H; ArH), 7.19 (d, ^4^*J*_H–H_ = 1.5 Hz, 4H; ArH), 7.29 (d, ^3^*J*_H–H_ = 7.7 Hz, 2H; ArH). (Accurate integration was not possible due to the presence of the minor isomer, 4-*cis*). ^13^C{^1^H} NMR (125 MHz, C_6_D_6_): *δ* (ppm) 24.17, 24.41, 24.45, 24.71, 24.81, 25.05, 25.10 (s; CH(*C*H_3_)_2_), 30.84, 30.86, 30.95, 31.01, 31.11, 34.88 (s; *C*H(CH_3_)_2_), 54.14 (br; N*C*H_2_Ph), 120.71, 120.80, 128.77, 129.01, 129.35, 130.55, 131.23, 131.38, 131.83, 132.05, 135.47, 137.05, 137.23, 139.80, 140.15, 140.54, 140.71, 141.54, 141.90, 146.77, 146.80, 146.86, 146.91, 148.49, 148.59 (s; ArC). ^31^P{^1^H} NMR (202 MHz, C_6_D_6_): *δ* (ppm) 535.4 (s, ^1^*J*_P–Se_ = 788.1 Hz; 4-*trans*), 528.6 (s; 4-*cis*). Ratio 4-*trans* : 4-*cis* = 4.81 : 1. ^77^Se NMR (95.4 MHz, d_8_-tol, −45 °C): *δ* (ppm) 1081.62 (d, ^1^*J*_P–Se_ = 875.0 Hz; 4-*trans*). HRMS (*m*/*z*): [M + H]^+^ calcd. For C_85_H_109_NPSe, 1254.7457; found 1254.7473.

## Author contributions

Conceptualization: C. H. and J. M. G.; experimental work: C. H.; X-ray crystallography: C. H. and M. P.; computational work: C. H.; writing – original draft: C. H. and J. M. G.; writing & editing: all authors; supervision: J. M. G.; funding acquisition: J. M. G.

## Conflicts of interest

There are no conflicts to declare.

## Supplementary Material

SC-OLF-D5SC04352B-s001

SC-OLF-D5SC04352B-s002

SC-OLF-D5SC04352B-s003

## Data Availability

The data supporting this article have been included as part of the ESI.†
