# Supplementary material for: Isolation of a phosphinidene sulfide and selenide
Source: Chem Sci. 2025 Jul 10;16(32):14690–7. doi: 10.1039/d5sc04352b (PMC12261337; doi:10.1039/d5sc04352b)
Supplement: SC-016-D5SC04352B-s002 [file SC-016-D5SC04352B-s002.pdf]

Supplementary Information (SI) for Chemical Science.
This journal is © The Royal Society of Chemistry 2025

Electronic Supplementary Information

Isolation of a Phosphinidene Sulfide and Selenide

Chenyang Hu,[a,b] Maren Pink,[a] and Jose M. Goicoechea[a,*]

[a] Department of Chemistry, Indiana University, 800 East Kirkwood Ave., Bloomington, Indiana, 47405, U.S.A.

[b] Department of Chemistry, University of Oxford, Chemistry Research Laboratory, 12 Mansfield Rd., Oxford, OX1 3TA, U.K.

E-mail: jgoicoec@iu.edu

Contents

1. Experimental details 2

2. NMR spectra 14

3. High Resolution Mass Spectrum 25

4. X-ray Crystallographic Data 27

6. References 45

1. Experimental details

1.1. General Synthetic Methods

All reactions and product manipulations were carried out under an inert atmosphere of argon or dinitrogen using standard Schlenk-line or glovebox techniques (MBraun UNIlab glovebox maintained at < 0.1 ppm H_2O and < 0.1 ppm O_2). Benzene (anhydrous, Sigma Aldrich), toluene (Fisher Chemical, HPLC grade), hexane (Fisher Chemical, HPLC grade) and acetonitrile (Fisher Chemical, HPLC grade) were purified using a Pure Process Technology (PPT) solvent purification system (SPS). C_6D_6 (Aldrich, 99.5%) and d_8-toluene (Aldrich, 99%) were distilled over sodium/benzophenone. $CDCl_3$ was distilled over CaH_2. All dry solvents were stored under argon in gas-tight ampoules over activated 3 Å molecular sieves. Compound **A** was synthesized following the procedure described in the literature.[1] Lawesson's reagent, Woollins' reagent and DMAP were purchased from TCI and Sigma Aldrich and used as received.

Additional characterization techniques: NMR spectra were acquired on a Bruker 500 MHz Avance Neo, a Varian 500 MHz Inova, or a Varian 400 MHz Inova NMR spectrometer. Chemical shifts (δ) are reported in parts per million (ppm). ^{1}H and ^{13}C NMR spectra are referenced to TMS using residual protio-solvent resonance (^{1}H NMR C_6D_6: δ = 7.16 ppm, ^{13}C NMR C_6D_6: δ = 128.06 ppm; ^{1}H NMR $CDCl_3$: δ = 7.26 ppm, ^{13}C NMR $CDCl_3$: δ = 77.16 ppm, ^{1}H NMR d_8-toluene: δ = 2.08 ppm). ^{31}P NMR spectra were externally referenced to an 85% solution of H_3PO_4 in H_2O. ^{77}Se NMR spectra were externally referenced to a solution of $SeMe_2$ in C_6D_6. High-resolution mass spectra were recorded on a Thermo Q-Exactive Plus (ESI-TOF, positive ion mode) instrument at the Mass Spectrometry Facility of the Department of Chemistry of Indiana University.

1.2. Synthesis of 1 from A.

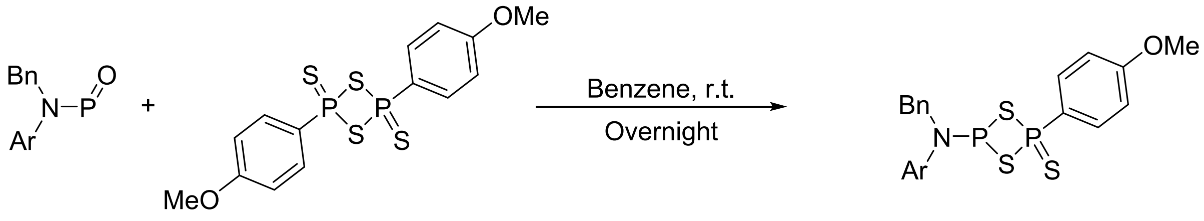

A Lawesson's reagent **1**

Scheme S1. Synthesis of **1** directly from **A.**

To a solid mixture of **A** (30 mg, 0.025 mmol, 1.00 eq.) and Lawesson's reagent (20 mg, 0.049 mmol, 2 eq.), benzene (0.5 mL) was added. The mixture was stirred overnight at room temperature and monitored by $^{31}P\{^{1}H\}$ NMR spectroscopy. Once compound **A** was fully consumed, the mixture was filtered, and MeCN (20 ml) was added to the filtrate. The mixture was left in a –35 °C freezer overnight to crystalize. The supernatant was removed, and the resulting solid was dried *in vacuo* to afford **1** (28 mg, 0.020 mmol; 79% yield) as a colorless powder. Single crystals of **1** suitable for X-ray diffraction were obtained by slow evaporation of a concentrated hexane solution of **1** at room temperature.

^{1}H NMR (500 MHz, C_6D_6): δ (ppm) 1.12 (d, $^{3}J_{H\text{–}H}$ = 6.8 Hz, 12H; CH(CH_3)$_2$), 1.15 (d, $^{3}J_{H\text{–}H}$ = 6.8 Hz, 18H; CH(CH_3)$_2$), 1.24–1.29 (m, 42H; CH(CH_3)$_2$), 2.85 (sept, $^{3}J_{H\text{–}H}$ = 6.8 Hz, 4H; C*H*(CH$_3$)$_2$), 3.04–3.10 (m, 8H; C*H*(CH$_3$)$_2$), 3.12 (s, 3H; OCH_3), 5.44 (s, 2H; NCH_2Ph), 6.59–6.62 (m, 2H; Ar*H*), 6.68–6.75 (m, 3H; Ar*H*), 7.00 (t, $^{3}J_{H\text{–}H}$ = 7.8 Hz, 1H; Ar*H*), 7.16 (s, overlapping with C_6D_6, 8H, Ar*H*), 7.17 (d, $^{4}J_{H\text{–}H}$ = 1.7 Hz, 2H; Ar*H*), 7.19 (d, $^{4}J_{P\text{–}H}$ = 1.7 Hz, 2H; Ar*H*), 7.22 (d, $^{4}J_{H\text{–}H}$ = 1.7 Hz, 2H; Ar*H*), 7.30 (d, $^{3}J_{H\text{–}H}$ = 7.8 Hz, 2H; Ar*H*), 7.56 (d, $^{3}J_{H\text{–}H}$ = 8.6 Hz, 2H; Ar*H*), 8.26 (dd, $^{3}J_{H\text{–}P}$ = 15.2 Hz $^{3}J_{H\text{–}H}$ = 8.6 Hz, 2H; Ar*H*). (Accurate integration was not possible as, in solution, **1** is in equilibrium with **2** and LR).

$^{13}C\{^1H\}$ NMR (125 MHz, C_6D_6): δ (ppm) 24.40, 24.43, 24.68, 25.04, 25.23, (s; $CH(\mathit{C}H_3)_2$) 30.71, 30.99, 31.11, 34.84, 34.93 (s; $\mathit{C}H(CH_3)_2$), 52.80 (br; $N\mathit{C}H_2Ph$) 54.88 (s; $O\mathit{C}H_3$), 113.81 (d, $^1J_{P–C}$ = 16.7 Hz), 120.89, 128.59, 129.63, 131.53, 131.72, 132.39, 133.49, 133.54, 133.61, 135.20 (d, $^1J_{P–C}$ = 17.1 Hz), 136.39, 137.07, 137.38, 140.28, 141.39, 141.90, 142.39 (d, $^2J_{P–C}$ = 6.6 Hz), 147.00, 148.53, 163.07 (s; Ar*C*).

^{31}P NMR (162 MHz, C_6D_6): δ (ppm) 42.48 (dt, $^3J_{P–P}$ = 93.2 Hz $^3J_{P–H}$ = 15.2 Hz; $PS\mathit{P}(S)C_6H_4OMe$), 47.84 (d, $^3J_{P–P}$ = 93.2 Hz; $\mathit{P}SP(S)C_6H_4OMe$).

$^{31}P\{^1H\}$ NMR (162 MHz, C_6D_6): δ (ppm) 42.48 (d, $^3J_{P–P}$ = 93.2 Hz; $PS\mathit{P}(S)C_6H_4OMe$), 47.84 (d, $^3J_{P–P}$ = 93.2 Hz; $\mathit{P}SP(S)C_6H_4OMe$).

HRMS (*m*/*z*): $[M+H]^+$ calcd. For $C_{92}H_{116}NOP_2S_3$, 1408.7689; found 1408.7670.

1.3. One-pot synthesis of 2

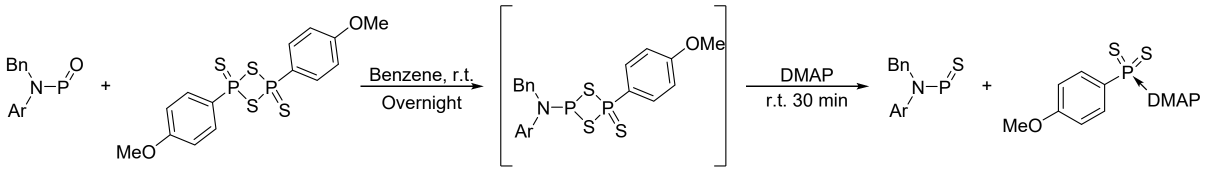

A Lawesson's reagent 1 2

Scheme S2. One-pot synthesis of **2**.

To a solid mixture of **A** (50 mg, 0.042 mmol, 1.00 eq.) and Lawesson's reagent (40 mg, 0.099 mmol, 2.3 eq.), benzene (0.7 mL) was added. The mixture was stirred overnight at room temperature and monitored by $^{31}P\{^1H\}$ NMR spectroscopy. Once compound **A** was fully consumed, the mixture was filtered, and DMAP (20 mg, 0.164 mmol, 3.9 eq.) was added to the filtrate. After stirring the mixture for 30 min at room temperature, the formation of a precipitate was observed. The mixture was filtered again, and MeCN (20 ml) was added to the filtrate. The mixture was left in a –35 °C freezer overnight to crystalize. The supernatant was removed and the resulting solid was dried *in vacuo* to afford **2** (42 mg, 0.035mmol; 83% yield) as a light-green powder. Single crystals of **2** suitable for X-ray diffraction were obtained by slow evaporation of a concentrated hexane solution of **2** at room temperature.

^1H NMR (500 MHz, C_6D_6): δ (ppm) 1.16 (d, $^3J_{H\text{–}H}$ = 6.8 Hz, 12H; CH(CH_3)$_2$), 1.20–1.22 (m, 24H; CH(CH_3)$_2$), 1.28–1.32 (m, 36H; CH(CH_3)$_2$), 2.87 (sept, $^3J_{H\text{–}H}$ = 6.8 Hz, 4H; CH(CH$_3$)$_2$), 2.93–3.06 (m, 8H; CH(CH$_3$)$_2$), 5.46 (s, 2H; NCH_2Ph), 6.61 (t, $^3J_{H\text{–}H}$ = 7.6 Hz, 2H; ArH), 6.74 (t, $^3J_{H\text{–}H}$ = 7.6 Hz, 2H; ArH), 6.79–6.83 (m, 1H; ArH), 6.95 (t, $^3J_{H\text{–}H}$ = 7.8 Hz, 1H; ArH), 7.10 (t, $^4J_{H\text{–}H}$ = 1.6 Hz, 2H; ArH), 7.16–7.17 (m, overlapping with C_6D_6, 8H; ArH), 7.19 (t, $^4J_{H\text{–}H}$ = 1.6 Hz, 2H; ArH), 7.21 (t, $^4J_{H\text{–}H}$ = 1.6 Hz, 2H; ArH), 7.31 (t, $^3J_{H\text{–}H}$ = 7.8 Hz, 2H; ArH). (Accurate integration was not possible due to the presence of the minor isomer, **2**-*cis*).

$^{13}C\{^1H\}$ NMR (125 MHz, C_6D_6): δ (ppm) 24.17, 24.37, 24.41, 24.45, 24.70, 24.81, 25.02,

25.09 (s; $CH(CH_3)_2$), 30.86, 30.89, 30.99, 31.00, 31.11, 34.88 (s; $CH(CH_3)_2$), 51.02 (br; NCH_2Ph), 120.72, 120.78, 128.59, 129.37, 130.53, 131.19, 131.37, 131.81, 131.91, 132.10, 135.78, 137.06, 137.24, 139.86, 140.61 (d, J_{P-C} = 5.2 Hz), 140.94, 141.57, 146.78, 146.90, 148.60 (s; Ar*C*).

$^{31}P\{^1H\}$ NMR (202 MHz, C_6D_6): δ (ppm) 476.97 (s; **2**-*trans*), 472.13 (s; **2**-*cis*). Ratio **2**-*trans*:**2**-*cis* = 4.28:1.

HRMS (*m*/*z*): $[M+H]^+$ calcd. For $C_{85}H_{109}NPS$, 1206.8013; found 1206.8017.

1.4. Synthesis of 1 from 2

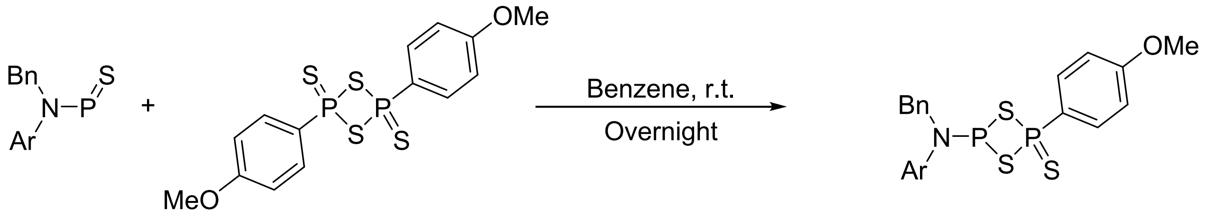

2 Lawesson's reagent **1**

Scheme S3. Synthesis of **1** from **2.**

To a solid mixture of **2** (10 mg, 0.0083 mmol, 1.00 eq.) and Lawesson's reagent (10 mg, 0.025 mmol, 3 eq.), benzene (0.5 mL) was added. The mixture was stirred overnight at room temperature and monitored by $^{31}P\{^{1}H\}$ NMR spectroscopy. Once compound **2** was fully consumed, the mixture was filtered, and MeCN (20 ml) was added to the filtrate. The mixture was left in a –35 °C freezer overnight to crystalize. The supernatant was removed and the resulting solid was dried *in vacuo* to afford **1** (9 mg, 0.0064mmol; 77% yield) as a colorless powder. All analytical data is identical to that described above in Section 1.2.

1.5. Independent synthesis of (MeO)$C_6H_4P(S)_2$(DMAP)

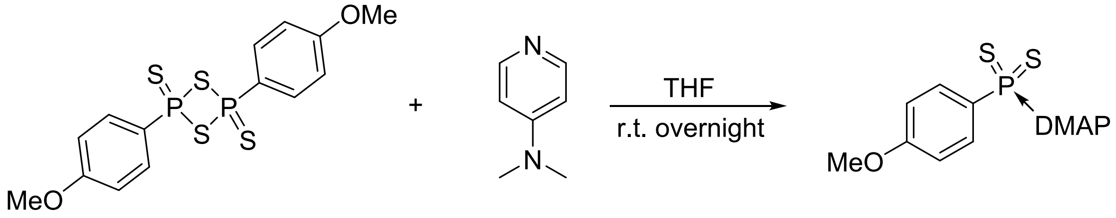

Scheme S4. Synthesis of (MeO)$C_6H_4P(S)_2$(DMAP).

To a solid mixture of Lawesson's reagent (20 mg, 0.049 mmol, 1.00 eq.) and DMAP (12 mg, 0.098 mmol, 2 eq.), THF (1 mL) was added. The mixture was stirred overnight at room temperature, after which all volatiles were removed *in vacuo*. The residual solid was washed with benzene three times (0.5 ml × 3), followed by three additional washes with hexane (0.5 ml × 3). The solid was dried under vacuum to afford (MeO)$C_6H_4P(S)_2$(DMAP) (24 mg, 0.074 mmol; 75% yield) as a colorless powder.

^{1}H NMR (500 MHz, $CDCl_3$): δ (ppm) 3.19 (s, 6H; N(CH_3)$_2$), 3.80 (s, 3H; OCH_3), 6.60 (d, $^{3}J_{H–H}$ = 7.9 Hz, 2H; Ar*H*), 6.85 (dd, $^{3}J_{H–H}$ = 9.1 Hz, $^{3}J_{H–P}$ = 2.6 Hz, 2H; Ar*H*), 8.10–8.15 (m, 2H; Ar*H*), 9.10–9.14 (m, 2H; Ar*H*).

^{13}C{^{1}H} NMR (125 MHz, $CDCl_3$): δ (ppm) 40.34 (s; N(*C*H_3)$_2$), 55.54 (s; O*C*H_3), 106.01 (d, $^{3}J_{C–P}$ = 4.0 Hz; Ar*C*), 113.41 (d, $^{2}J_{C–P}$ = 15.5 Hz; Ar*C*), 132.76 (d, $^{2}J_{C–P}$ = 14.5 Hz; Ar*C*), 132.98 (d, $^{1}J_{C–P}$ = 101.5 Hz; Ar*C*), 141.12 (d, $^{3}J_{C–P}$ = 6.0 Hz; Ar*C*), 156.46 (s; Ar*C*), 161.96 (d, $^{4}J_{C–P}$ = 3.1 Hz; Ar*C*).

^{31}P NMR (202 MHz, $CDCl_3$): δ (ppm) 109.44 (s).

^{31}P{^{1}H} NMR (202 MHz, $CDCl_3$): δ (ppm) 109.44 (s).

1.6. Synthesis of 3 from A

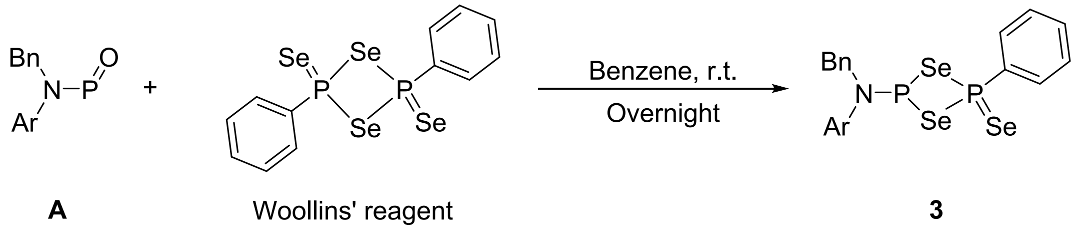

Scheme S5. Synthesis of **3** from **A.**

To a solid mixture of **A** (30 mg, 0.025 mmol, 1.00 eq.) and Woollins' reagent (26 mg, 0.049 mmol, 2 eq.), benzene (0.5 mL) was added. The mixture was stirred overnight at room temperature and monitored by $^{31}P\{^{1}H\}$ NMR spectroscopy. Once compound **A** was fully consumed, the mixture was filtered, and MeCN (20 ml) was added to the filtrate. The mixture was left in a –35 °C freezer overnight to crystalize. The supernatant was removed and the resulting solid was dried *in vacuo* to afford **3** (31 mg, 0.020 mmol; 82% yield) as a bright-yellow powder. Single crystals of **3** suitable for X-ray diffraction were obtained by slow evaporation of a concentrated hexane solution of **3** under room temperature.

^{1}H NMR (500 MHz, C_6D_6): δ (ppm) 1.11 (d, $^{3}J_{H–H}$ = 6.9 Hz, 18H; CH(CH_3)$_2$), 1.17 (d, $^{3}J_{H–H}$ = 6.9 Hz, 18H; CH(CH_3)$_2$), 1.24–1.30 (m, 36H; CH(CH_3)$_2$), 2.85 (sept, $^{3}J_{H–H}$ = 6.9 Hz, 4H; CH(CH$_3$)$_2$), 3.01–3.14 (m, 8H; CH(CH$_3$)$_2$), 5.53 (s, 2H; NCH_2Ph), 6.69–6.75 (m, 3H; ArH), 6.97–7.02 (m, 3H; ArH), 7.16 (s, overlapping with C_6D_6, 8H; ArH), 7.18 (d, $^{4}J_{H–H}$ = 1.7 Hz, 2H; ArH), 7.20 (d, $^{4}J_{H–H}$ = 1.7 Hz, 2H; ArH), 7.25 (t, $^{4}J_{H–H}$ = 1.7 Hz, 2H; ArH), 7.29 (d, $^{3}J_{H–H}$ = 7.7 Hz, 2H; ArH), 7.53–7.58 (m, 3H; ArH), 8.42 (dd, $^{3}J_{H–P}$ = 17.0 Hz $^{3}J_{H–H}$ = 7.2 Hz, 2H; ArH). (Accurate integration was not possible as, in solution, **3** is in equilibrium with **4** and WR).

$^{13}C\{^{1}H\}$ NMR (125 MHz, C_6D_6): δ (ppm) 24.40, 24.44, 24.68, 25.04, 25.32 (s; CH(CH_3)$_2$), 30.77, 30.84, 30.90, 31.05, 31.11, 34.84, 34.88 (s; CH(CH$_3$)$_2$), 55.24 (br; NCH$_2$Ph), 120.71,

120.82, 120.96, 128.59, 129.63, 131.46, 131.56, 131.62, 131.82, 132.39, 136.26, 136.95, 137.05, 137.17, 137.38, 138.87 (d, $^{1}J_{C–P}$ = 25.3 Hz), 140.13, 140.88, 141.40, 141.54, 141.90, 142.40 (d, $^{2}J_{C–P}$ = 6.2 Hz) 143.84 (br), 146.79, 146.91, 146.96, 147.05, 147.10, 148.56, 148.63 (s; Ar*C*).

^{31}P NMR (202 MHz, C_6D_6): δ (ppm) –45.95 (dt, $^{3}J_{P–P}$ = 80.6 Hz $^{3}J_{P–H}$ = 17.0 Hz; PSe*P*(Se)Ph), 46.04 (d, $^{3}J_{P–P}$ = 80.6 Hz, *P*SeP(Se)Ph).

^{31}P{^{1}H} NMR (202 MHz, C_6D_6): δ (ppm) –45.95 (d, $^{3}J_{P–P}$ = 80.6 Hz; PSe*P*(Se)Ph), 46.04 (d, $^{3}J_{P–P}$ = 80.6 Hz; *P*SeP(Se)Ph).

HRMS (*m*/*z*): $[M+H]^+$ calcd. For $C_{91}H_{114}NP_2Se_3$, 1520.5925; found 1520.5954.

1.7. One-pot synthesis of 4

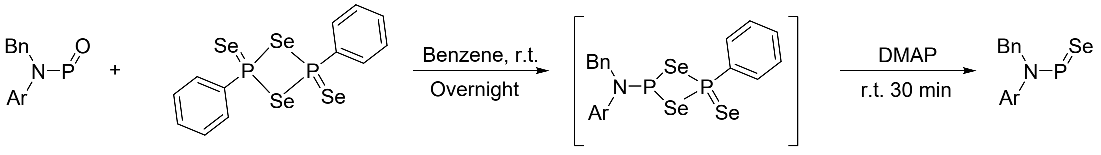

A Woollins' reagent **3** **4**

Scheme S6. One-pot synthesis of **4**.

To a solid mixture of **A** (50 mg, 0.042 mmol, 1.00 eq.) and Woollins' reagent (40 mg, 0.075 mmol, 1.8 eq.), benzene (0.7 mL) was added. The mixture was stirred overnight at room temperature and monitored by $^{31}P\{^{1}H\}$ NMR spectroscopy. Once compound **A** was fully consumed, the mixture is filtered, and DMAP (20 mg, 0.164 mmol, 3.9 eq.) was added to the filtrate. After stirring the mixture for 30 min at room temperature, the formation of a precipitate was observed. The mixture was filtered again, and MeCN (20 ml) was added to the filtrate. The mixture was left in a –35 °C freezer overnight to crystalize. The supernatant was removed and the resulting solid was dried *in vacuo* to afford **4** (42 mg, 0.033mmol; 80% yield) as a light-red powder. Single crystals of **4** suitable for X-ray diffraction were obtained by slow evaporation of a concentrated hexane solution of **4** under room temperature.

^{1}H NMR (500 MHz, C_6D_6): δ (ppm) 1.15 (d, $^{3}J_{H\text{–}H}$ = 6.8 Hz, 12H; CH(CH_3)$_2$), 1.18 (d, $^{3}J_{H\text{–}H}$ = 6.8 Hz, 12H; CH(CH_3)$_2$), 1.21 (d, $^{3}J_{H\text{–}H}$ = 6.8 Hz, 12H; CH(CH_3)$_2$), 1.28 (d, $^{3}J_{H\text{–}H}$ = 6.8 Hz, 24H; CH(CH_3)$_2$), 1.30–1.32 (m, 12H; CH(CH_3)$_2$), 2.86 (sept, $^{3}J_{H\text{–}H}$ = 6.8 Hz, 4H; CH(CH$_3$)$_2$), 2.92–3.07 (m, 8H; CH(CH$_3$)$_2$), 5.49 (s, 2H; NCH_2Ph), 6.61 (t, $^{3}J_{H\text{–}H}$ = 7.5 Hz, 2H; ArH), 6.72–6.75 (m, 2H; ArH), 6.79–6.81 (m, 1H; ArH), 6.94 (t, $^{3}J_{H\text{–}H}$ = 7.7 Hz, 1H; ArH), 7.09 (t, $^{4}J_{H\text{–}H}$ = 1.5 Hz, 2H; ArH), 7.16–7.17 (m, overlapping with C_6D_6, 8H; ArH), 7.19 (d, $^{4}J_{H\text{–}H}$ = 1.5 Hz, 4H; ArH), 7.29 (d, $^{3}J_{H\text{–}H}$ = 7.7 Hz, 2H; ArH). (Accurate integration was not possible due to the presence of the minor isomer, **4**-*cis*).

$^{13}C\{^1H\}$ NMR (125 MHz, C_6D_6): δ (ppm) 24.17, 24.41, 24.45, 24.71, 24.81, 25.05, 25.10 (s; $CH(CH_3)_2$), 30.84, 30.86, 30.95, 31.01, 31.11, 34.88 (s; $CH(CH_3)_2$), 54.14 (br; NCH_2Ph), 120.71, 120.80, 128.77, 129.01, 129.35, 130.55, 131.23, 131.38, 131.83, 132.05, 135.47, 137.05, 137.23, 139.80, 140.15, 140.54, 140.71, 141.54, 141.90, 146.77, 146.80, 146.86, 146.91, 148.49, 148.59 (s; Ar*C*).

$^{31}P\{^1H\}$ NMR (202 MHz, C_6D_6): δ (ppm) 535.42 (s, $^1J_{P-Se}$ = 788.1 Hz; **4**-*trans*), 528.60 (s; **4**-*cis*). Ratio **4**-*trans*:**4**-*cis* = 4.81:1.

^{77}Se NMR (95.4 MHz, d_8-tol, –45 °C): δ (ppm) 1081.62 (d, $^1J_{P-Se}$ = 875.03 Hz; **4**-*trans*).

HRMS (*m*/*z*): $[M+H]^+$ calcd. For $C_{85}H_{109}NPSe$, 1254.7457; found 1254.7473.

1.8. Synthesis of 3 from 4

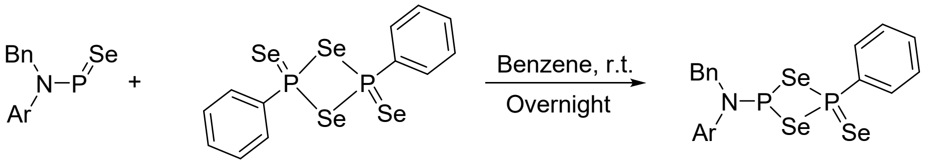

4 Woollins' reagent **3**

Scheme S7. Synthesis of **3** from **4.**

To a solid mixture of **4** (10 mg, 0.0080 mmol, 1.00 eq.) and Woollins' reagent (10 mg, 0.019 mmol, 2.3 eq.), benzene (0.5 mL) was added. The mixture was stirred overnight at room temperature and monitored by $^{31}P\{^{1}H\}$ NMR spectroscpy. Once compound **4** was fully consumed, the mixture was filtered, and MeCN (20 ml) was added to the filtrate. The mixture was left in a –35 °C freezer overnight to crystalize. The supernatant was removed and the resulting solid was dried *in vacuo* to afford **3** (9 mg, 0.0059 mmol; 74% yield) as a bright-yellow powder. All analytical data are identical to that reported in Section 1.6.

2. NMR spectra

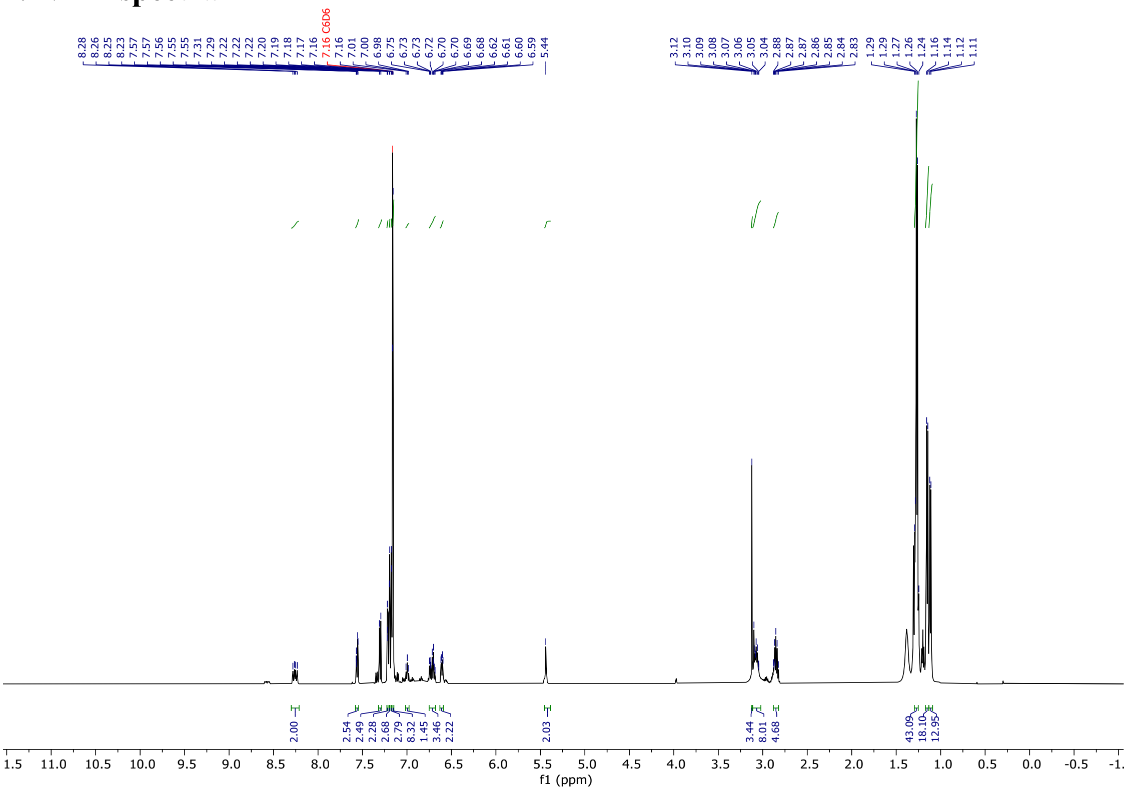

Figure S1. ^{1}H NMR spectrum of **1** (500 MHz, C_6D_6).

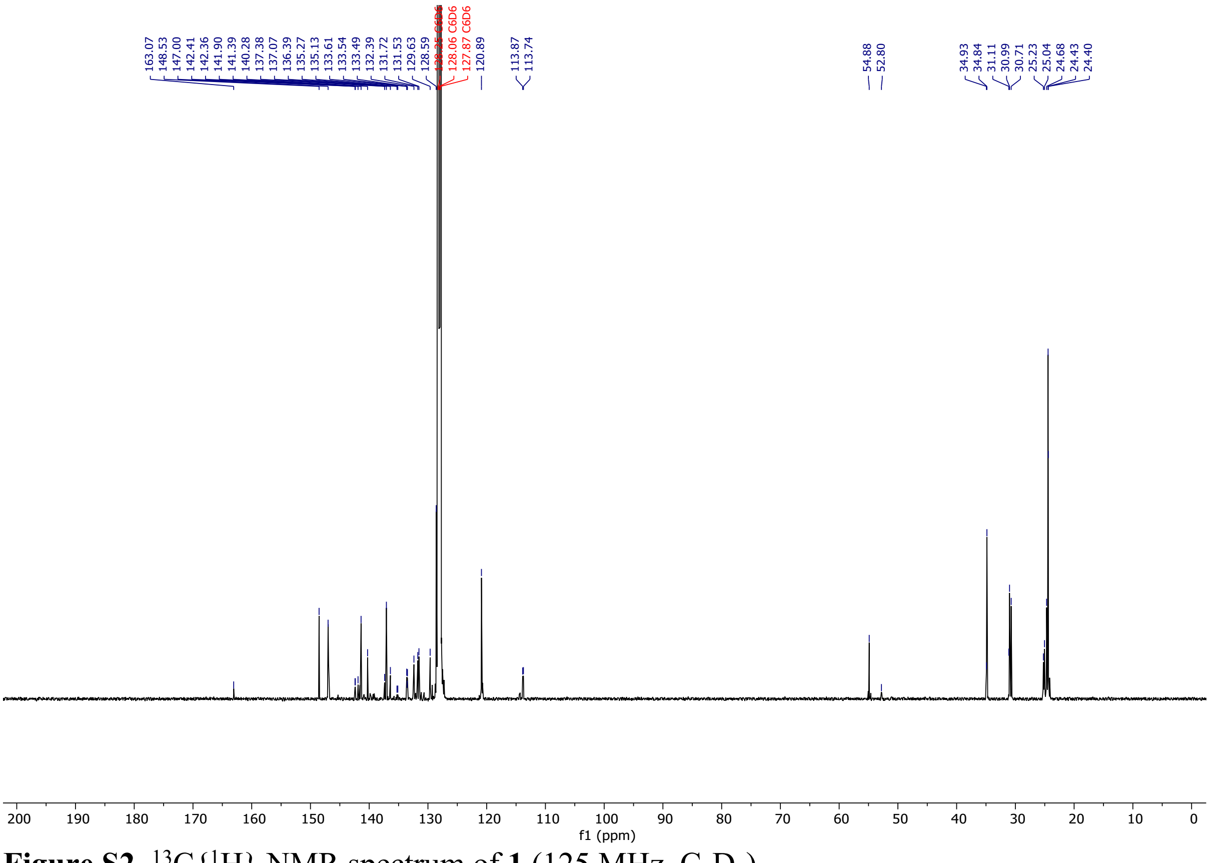

Figure S2. $^{13}C\{^{1}H\}$ NMR spectrum of **1** (125 MHz, C_6D_6).

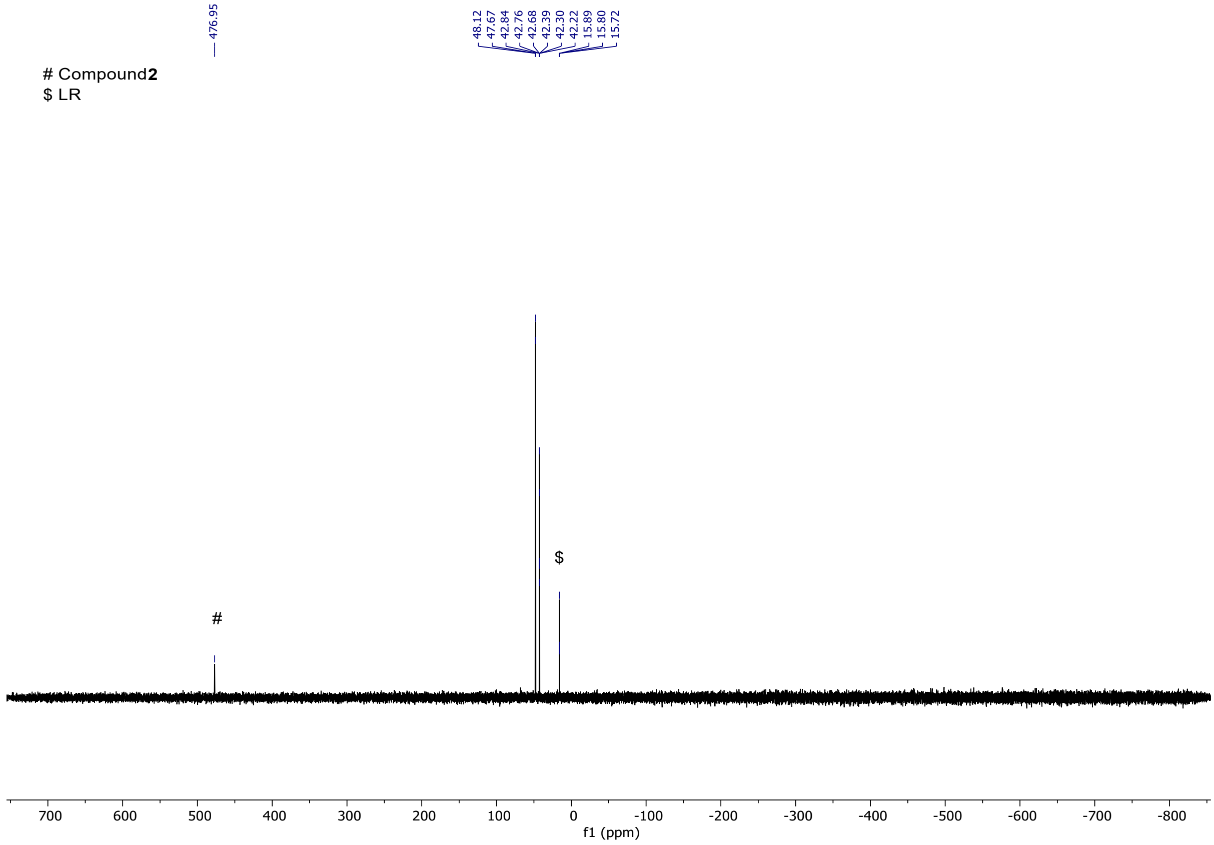

Figure S3. ^{31}P NMR spectrum of **1** (202 MHz, C_6D_6).

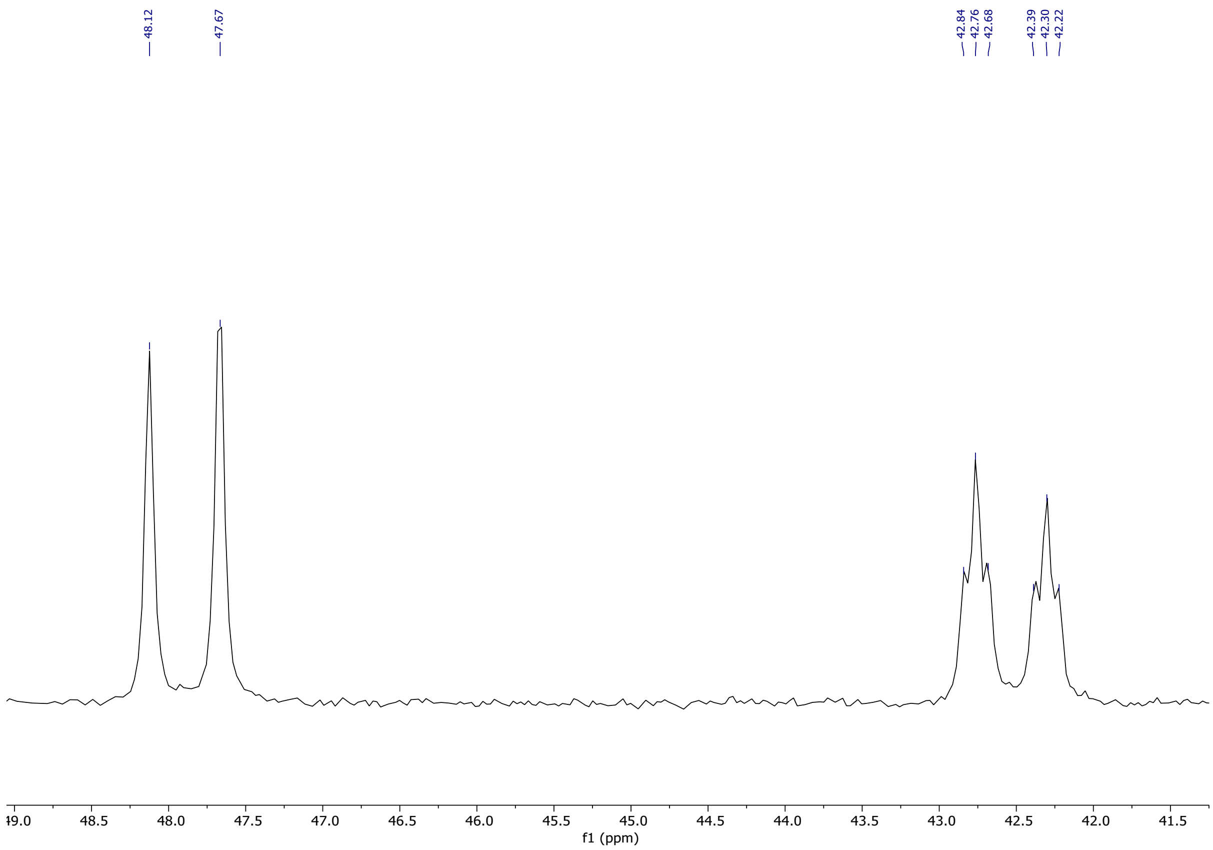

Figure S4. Zoomed in ^{31}P NMR spectrum of **1** (202 MHz, C_6D_6).

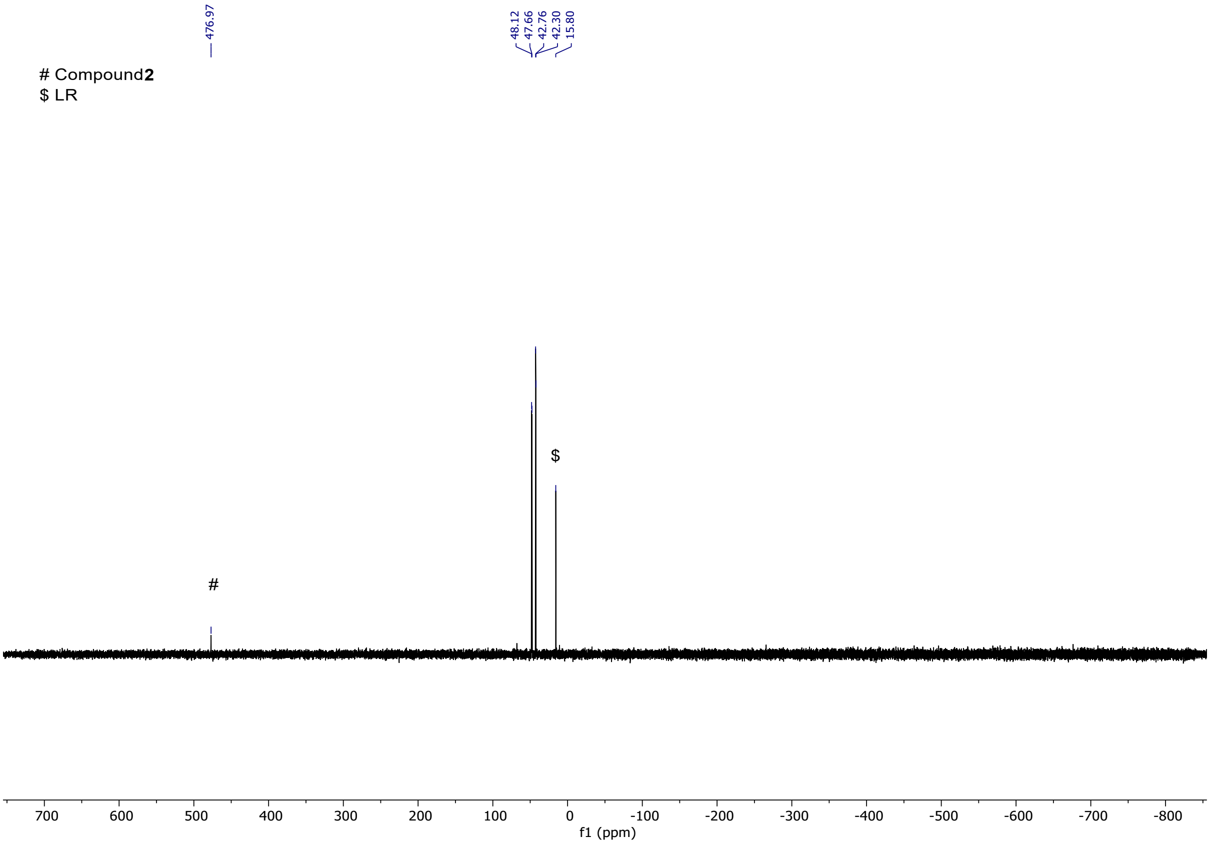

Figure S5. $^{31}P\{^{1}H\}$ NMR spectrum of **1** (202 MHz, C_6D_6).

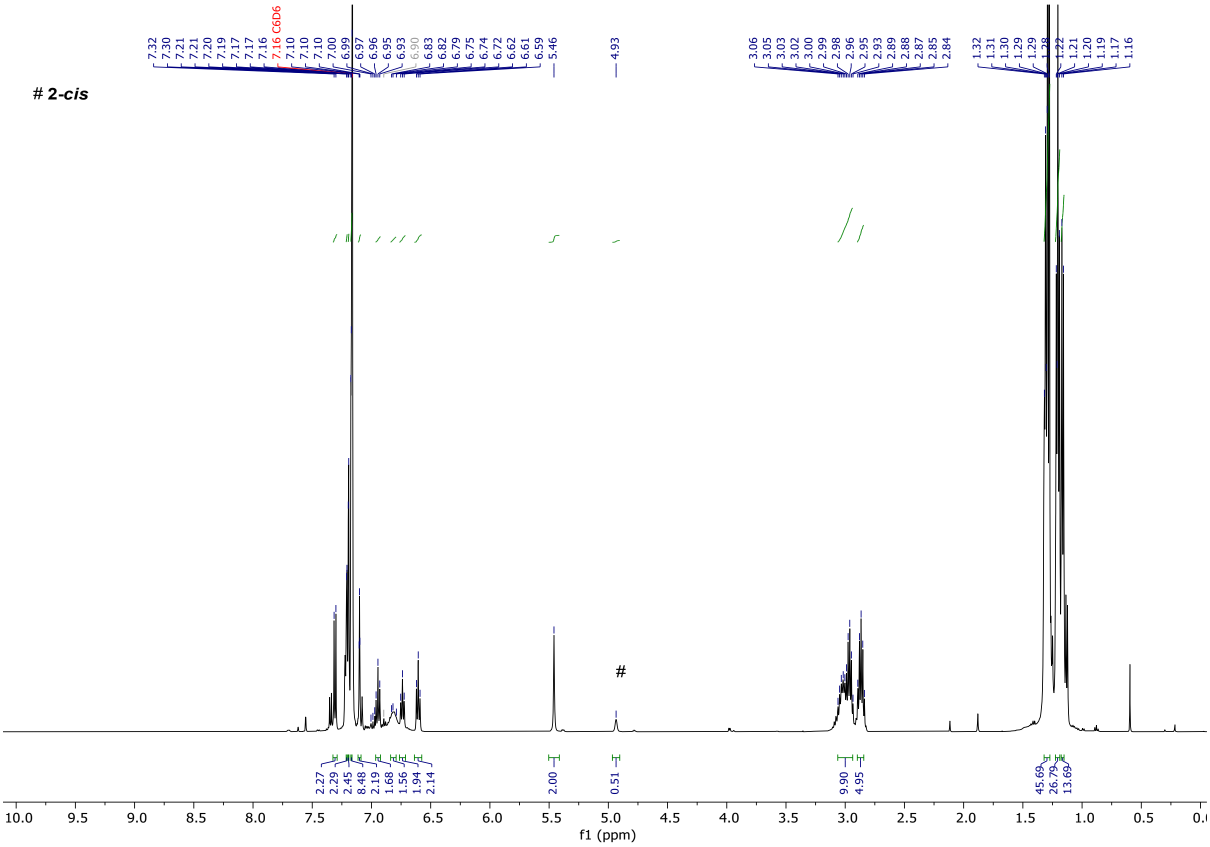

Figure S6. ^{1}H NMR spectrum of **2** (500 MHz, C_6D_6).

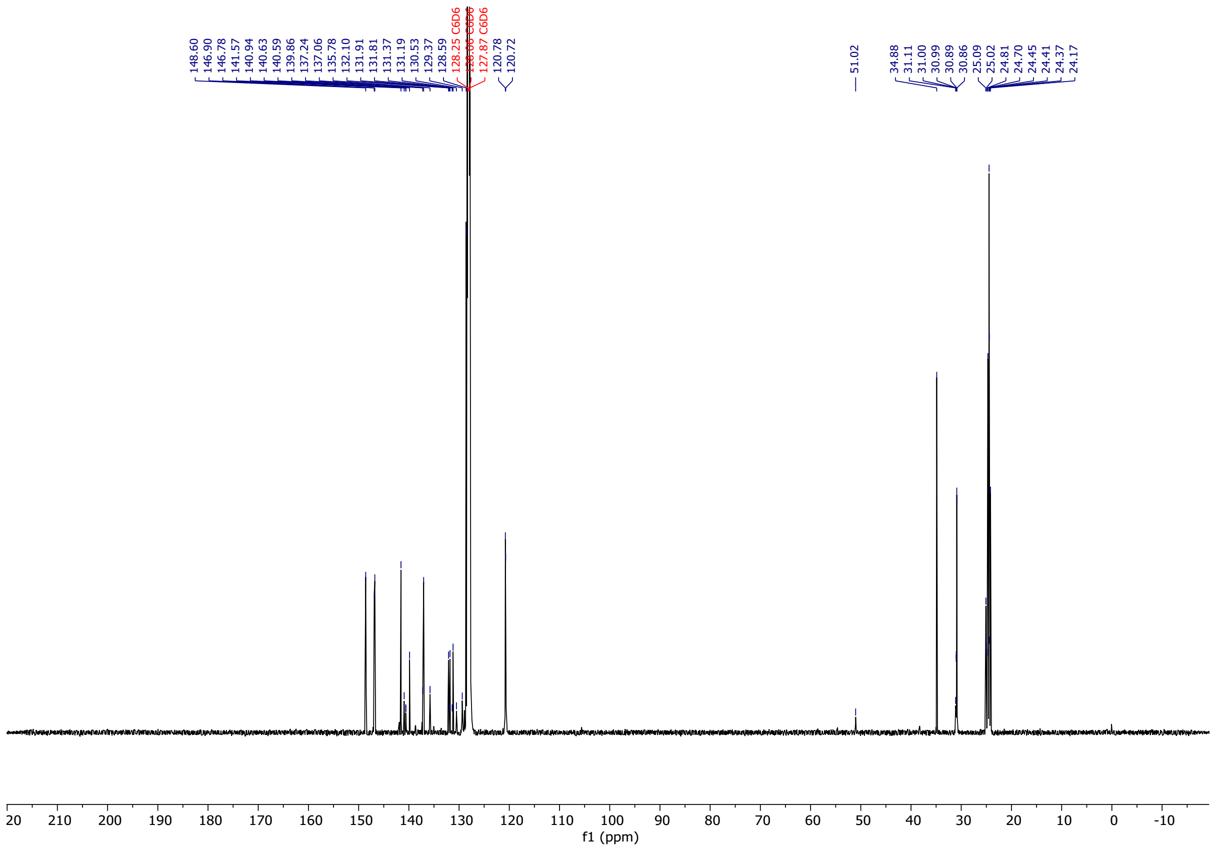

Figure S7. $^{13}C\{^{1}H\}$ NMR spectrum of **2** (125 MHz, C_6D_6).

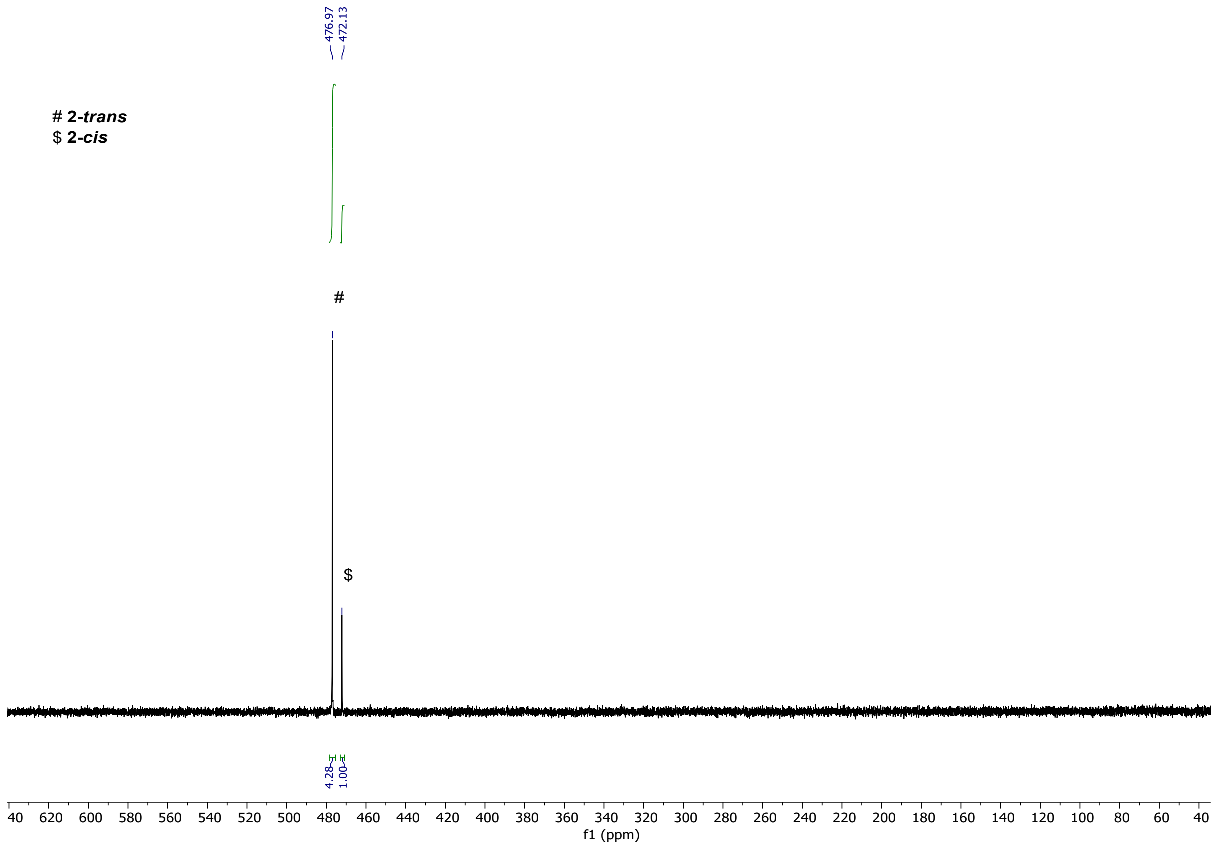

Figure S8. $^{31}P\{^{1}H\}$ NMR spectrum of **2** (202 MHz, C_6D_6).

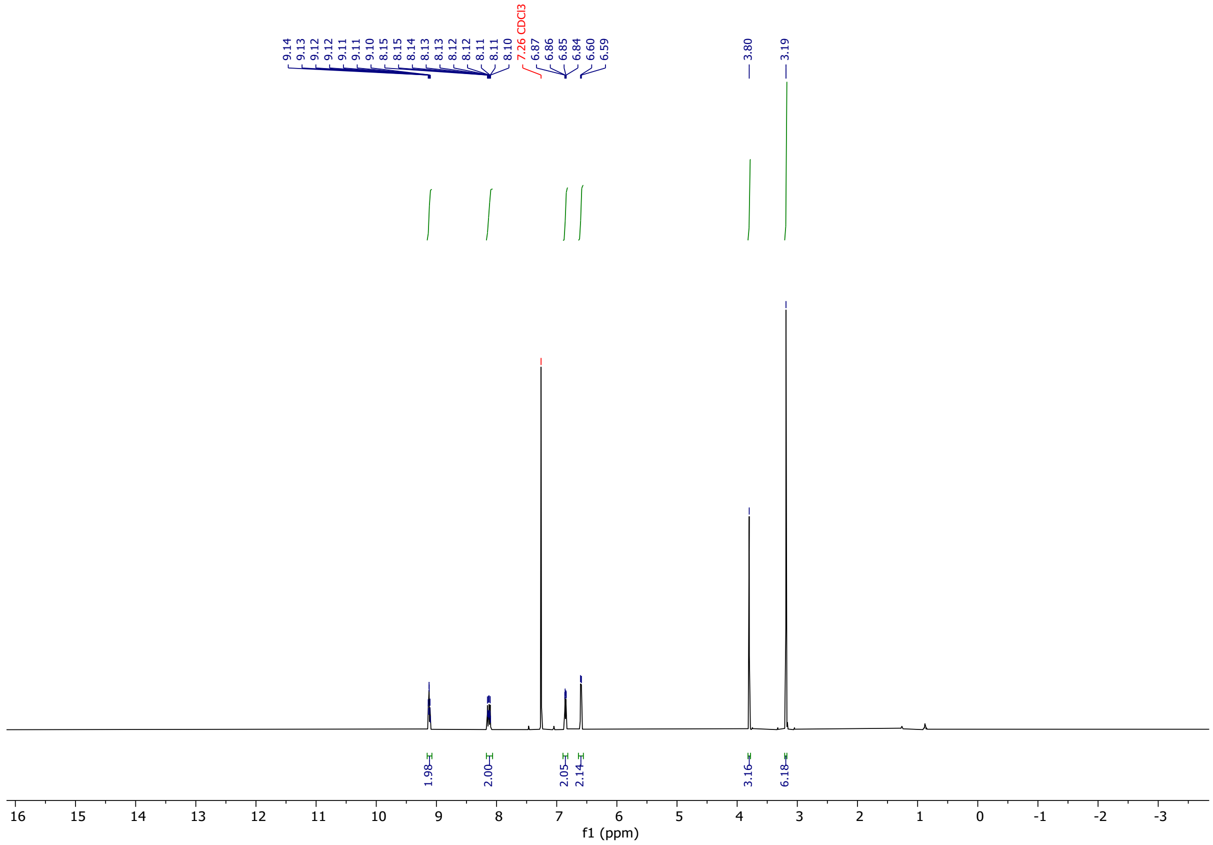

Figure S9. ^{1}H NMR spectrum of $(MeO)C_6H_4P(S)_2(DMAP)$ (500 MHz, $CDCl_3$).

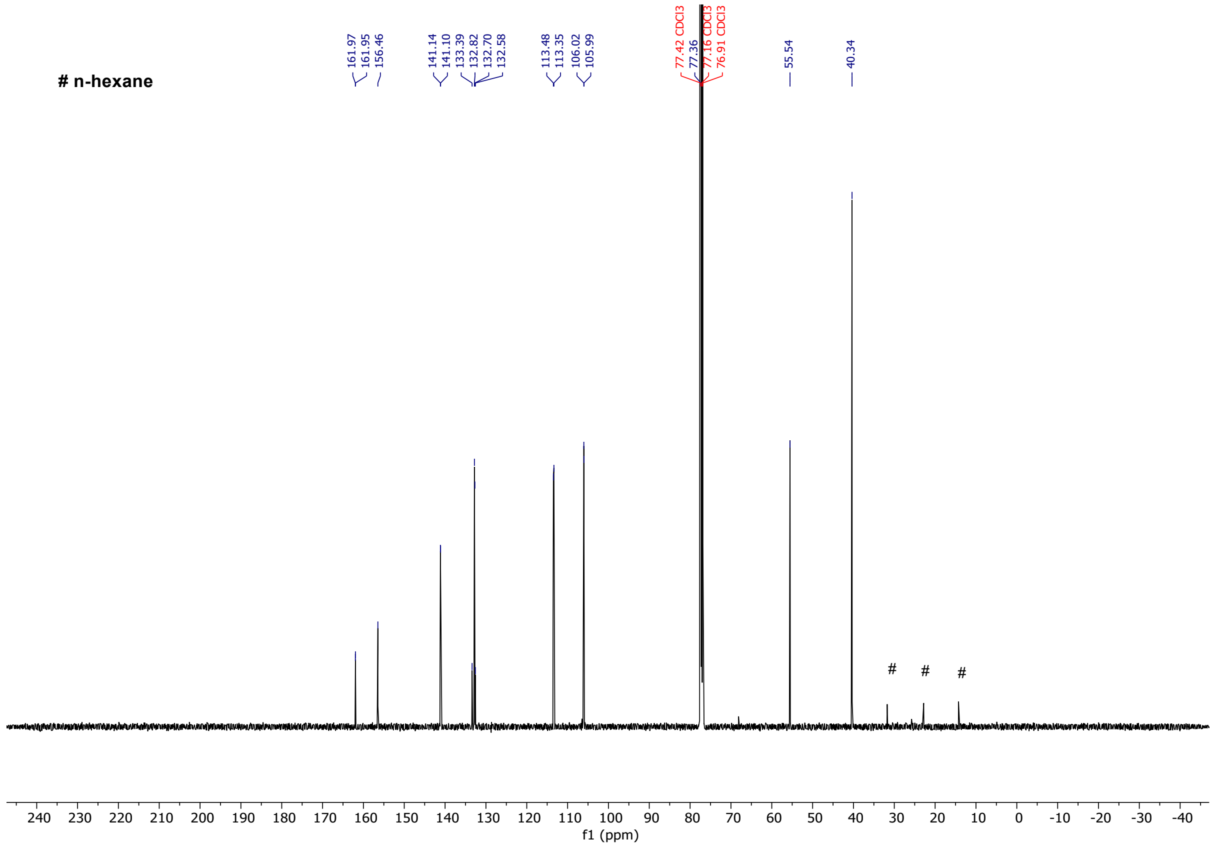

Figure S10. $^{13}C\{^{1}H\}$ NMR spectrum of $(MeO)C_6H_4P(S)_2(DMAP)$ (125 MHz, $CDCl_3$).

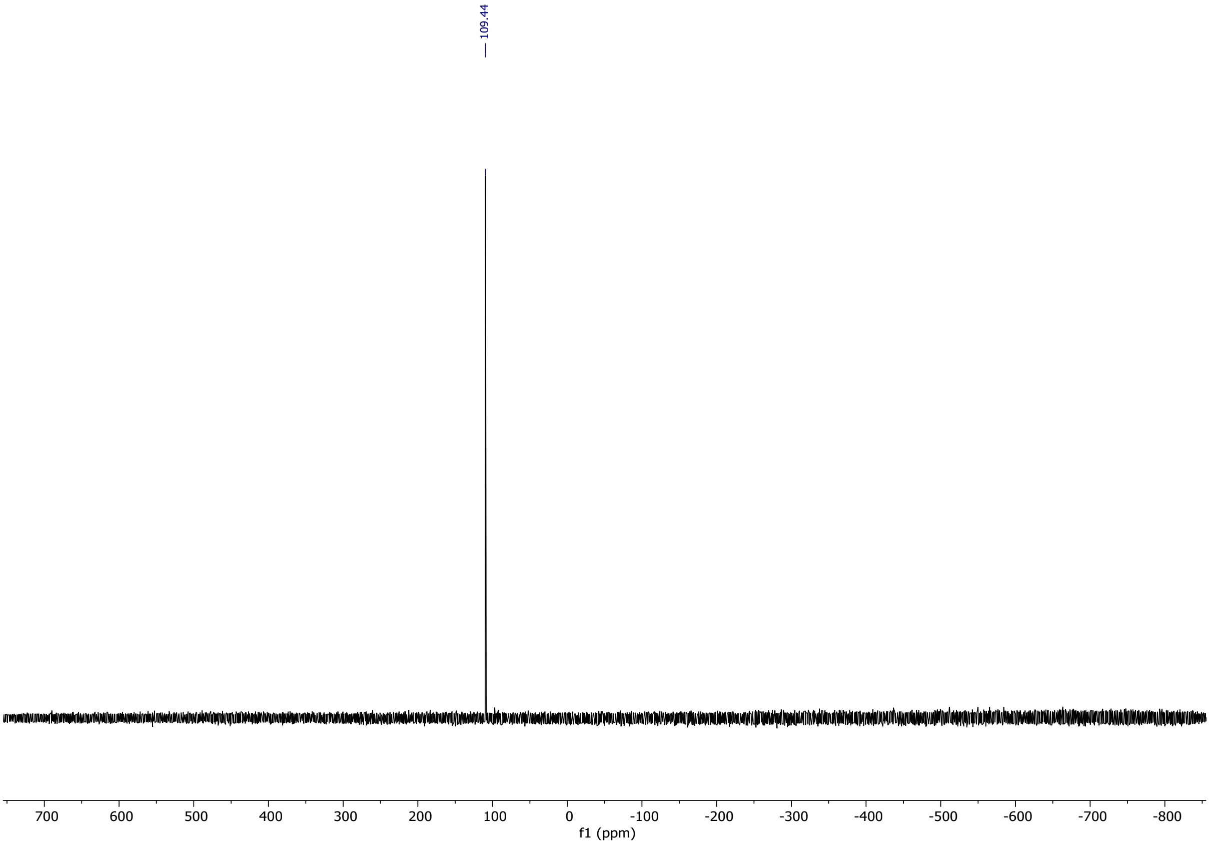

Figure S11. ^{31}P NMR spectrum of (MeO)$C_6H_4P(S)_2$(DMAP) (202 MHz, $CDCl_3$).

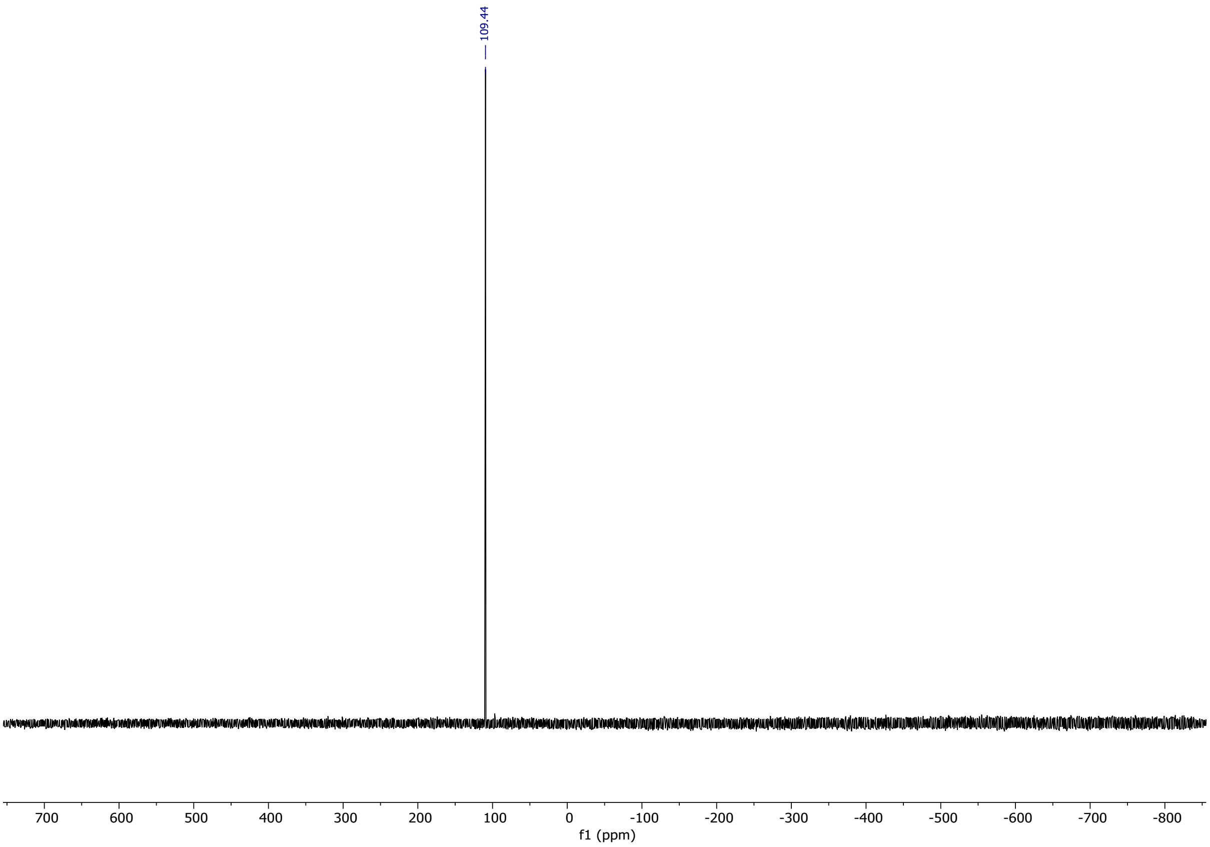

Figure S12. $^{31}P\{^1H\}$ NMR spectrum of (MeO)$C_6H_4P(S)_2$(DMAP) (202 MHz, $CDCl_3$).

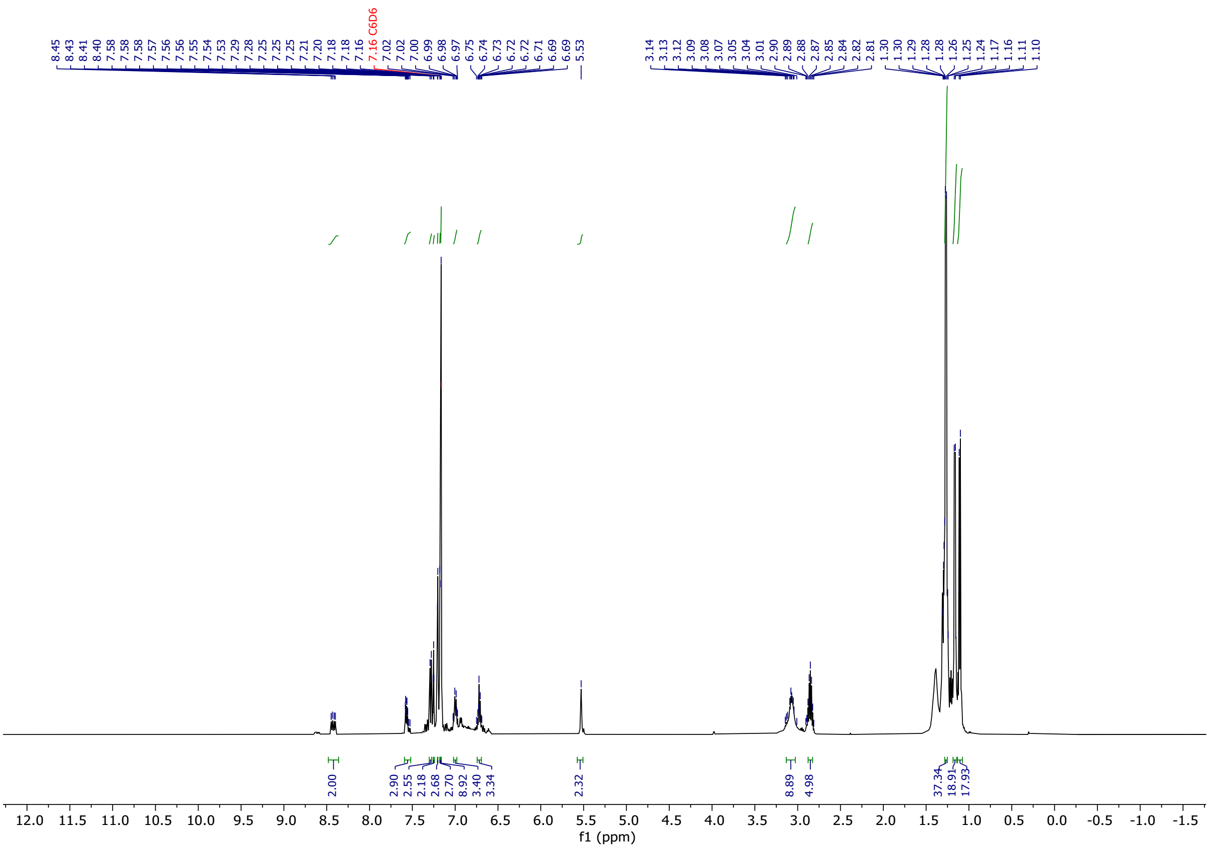

Figure S13. ^{1}H NMR spectrum of **3** (500 MHz, C_6D_6).

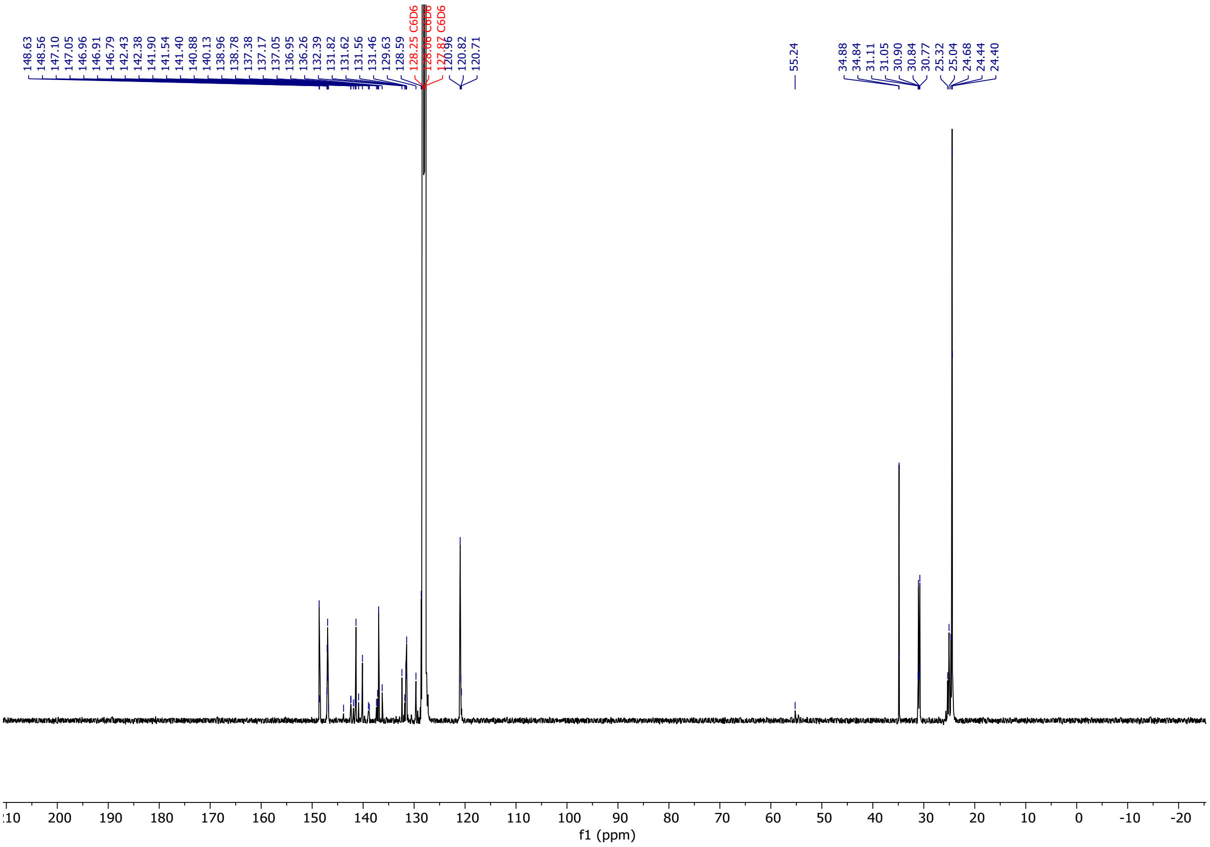

Figure S14. $^{13}C\{^{1}H\}$ NMR spectrum of **3** (125 MHz, C_6D_6).

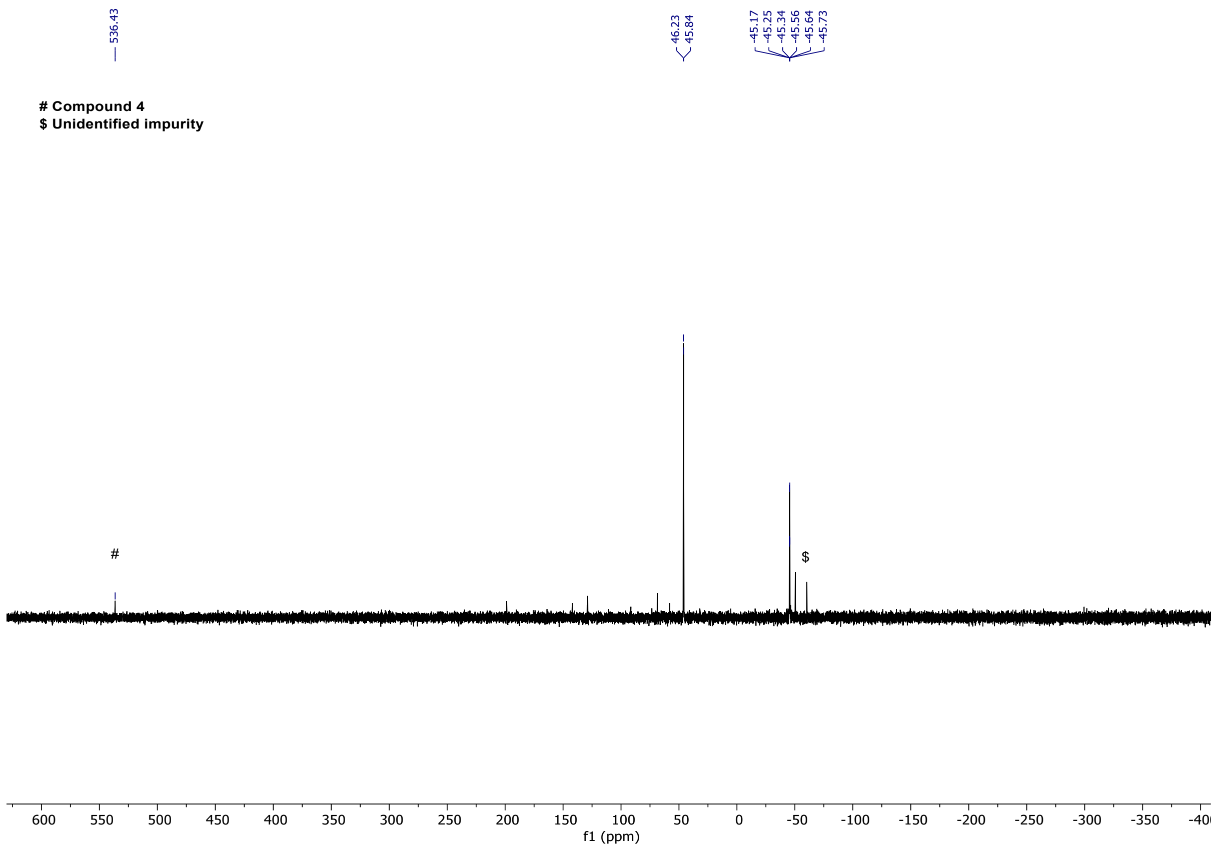

Figure S15. ^{31}P NMR spectrum of **3** (202 MHz, C_6D_6).

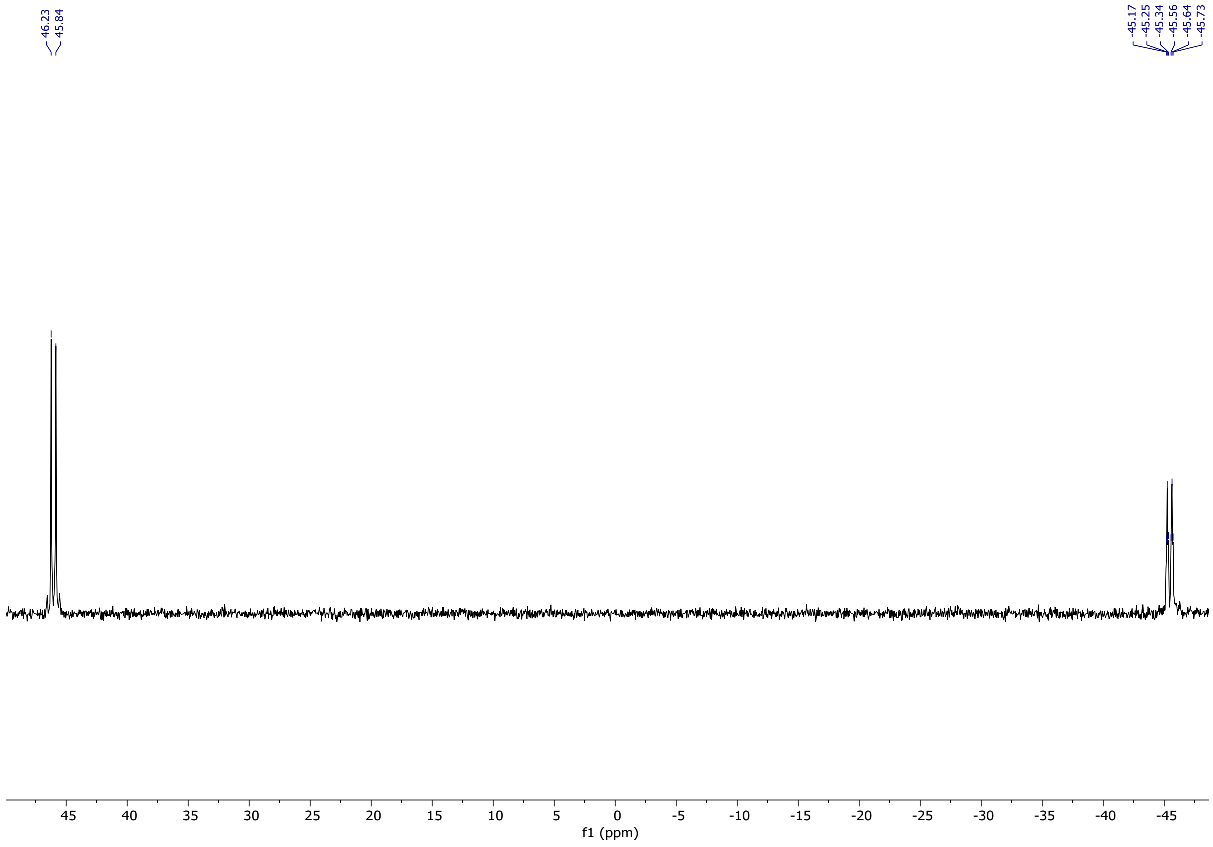

Figure S16. Zoomed in ^{31}P NMR spectrum of **3** (202 MHz, C_6D_6).

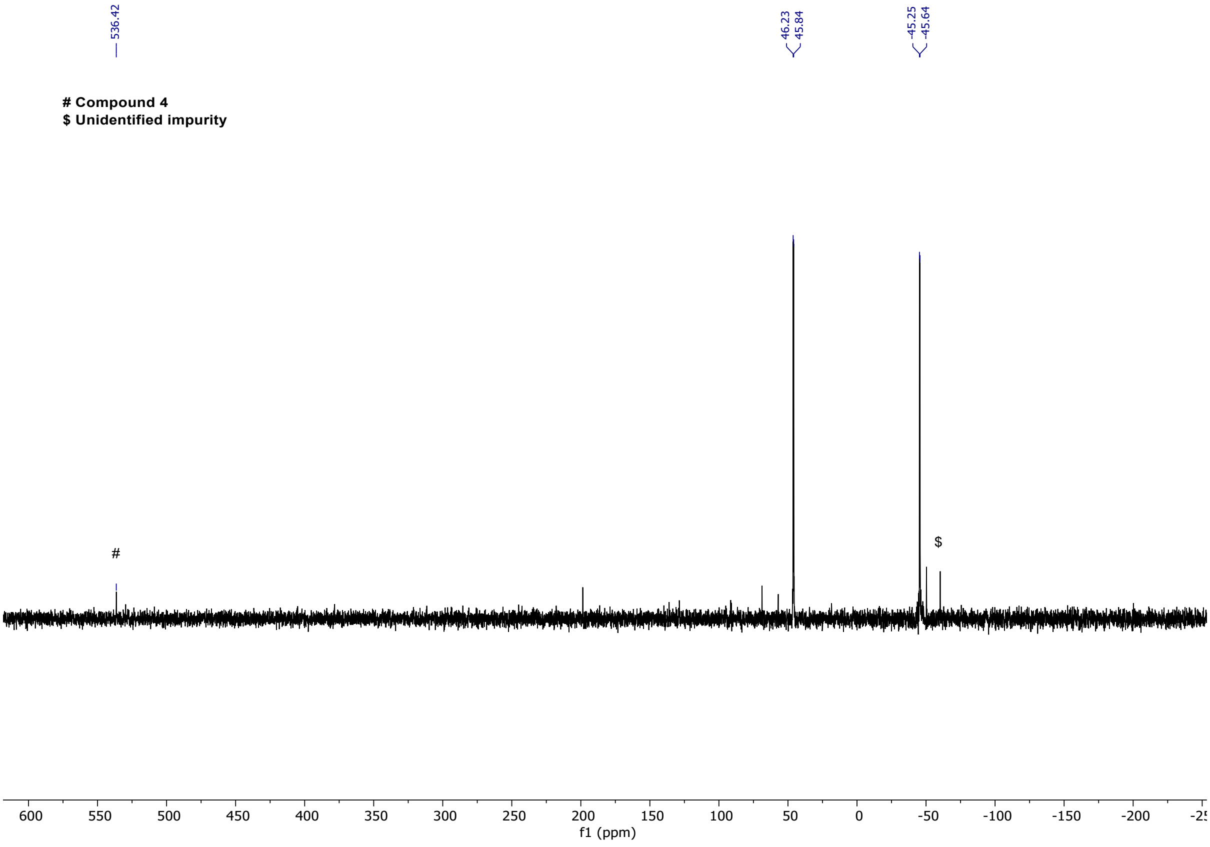

Figure S17. $^{31}P\{^{1}H\}$ NMR spectrum of **3** (202 MHz, C_6D_6).

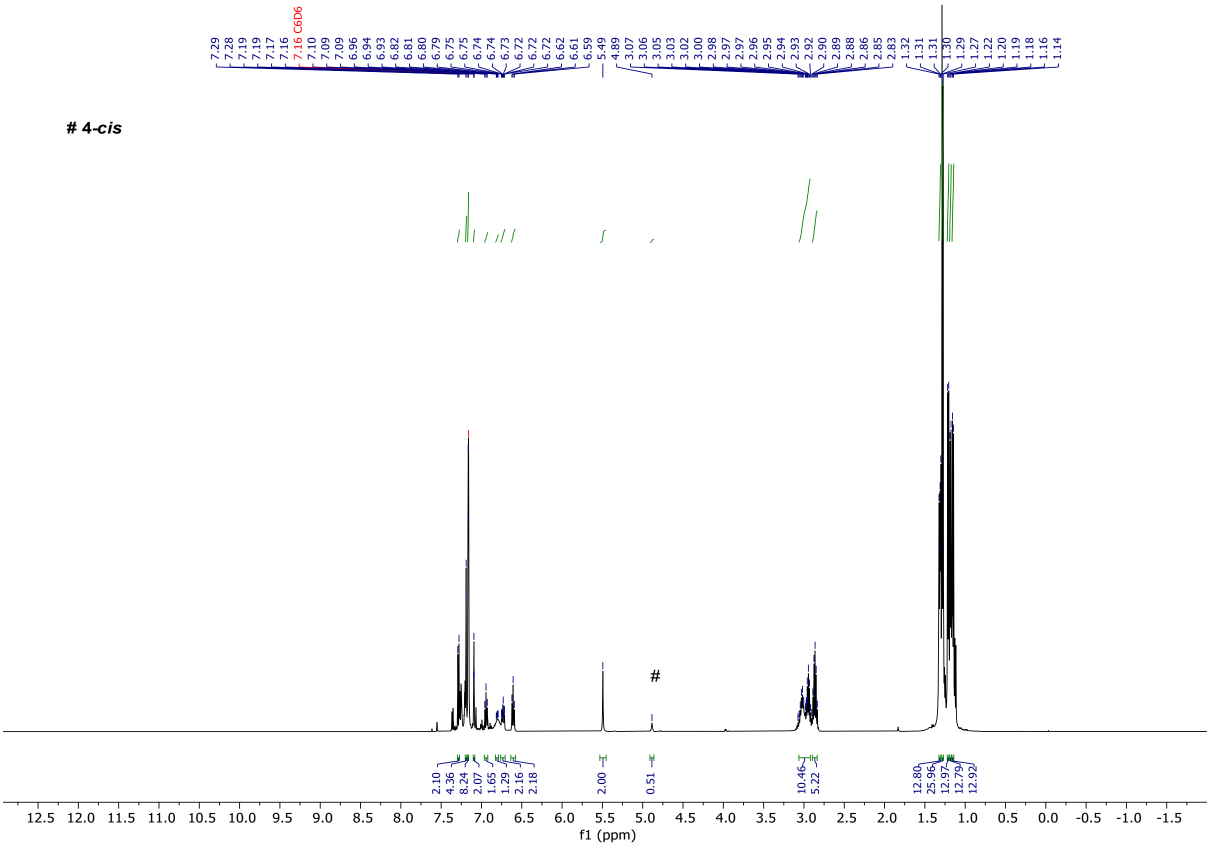

Figure S18. ^{1}H NMR spectrum of **4** (500 MHz, C_6D_6).

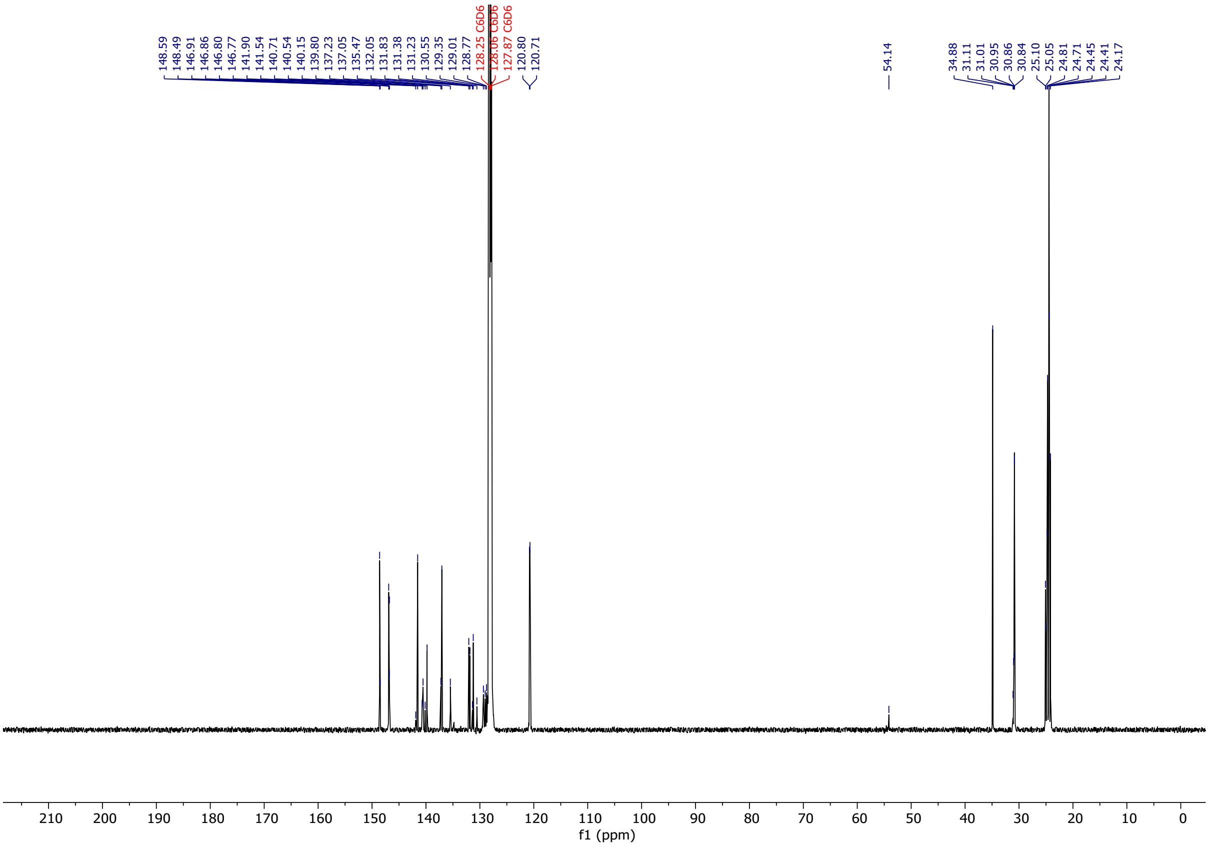

Figure S19. $^{13}C\{^{1}H\}$ NMR spectrum of **4** (125 MHz, C_6D_6).

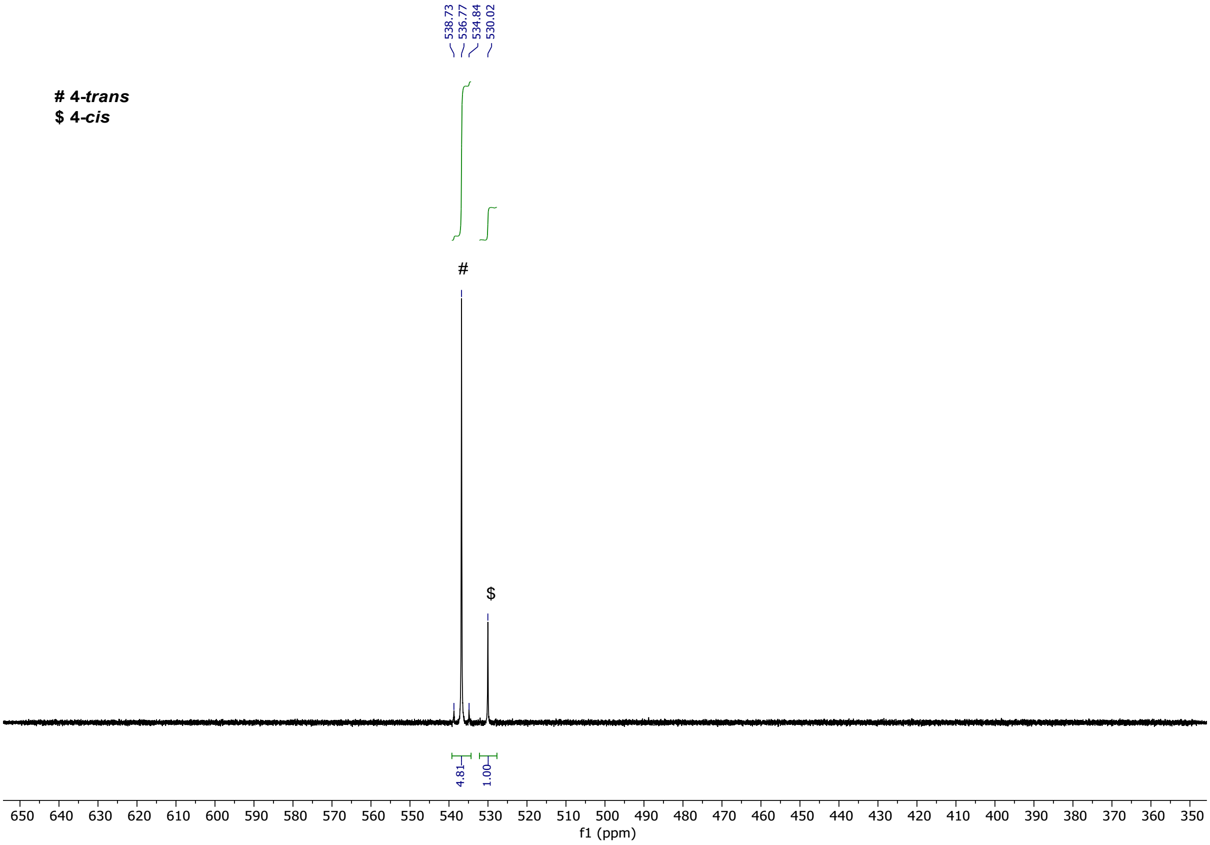

Figure S20. $^{31}P\{^{1}H\}$ NMR spectrum of **4** (202 MHz, C_6D_6).

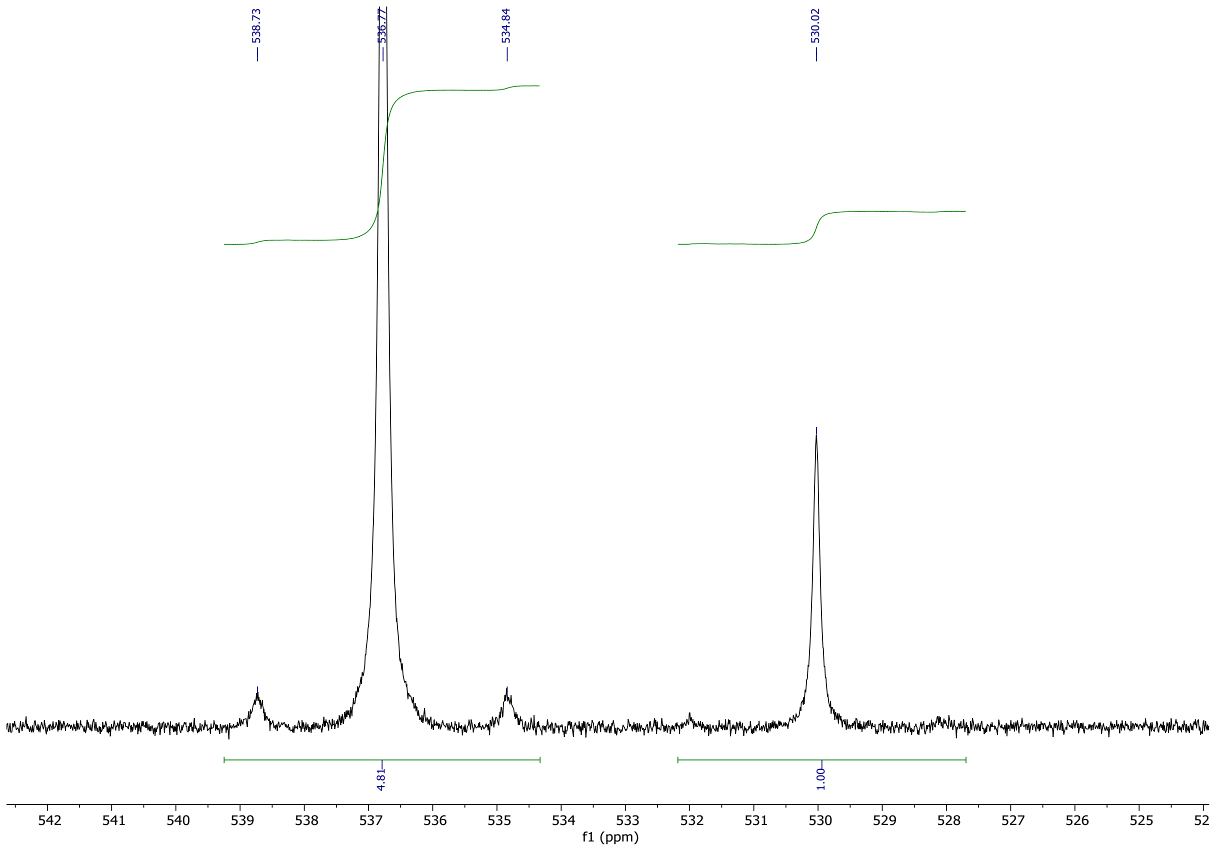

Figure S21. Zoomed in $^{31}P\{^{1}H\}$ NMR spectrum of **4** (202 MHz, C_6D_6).

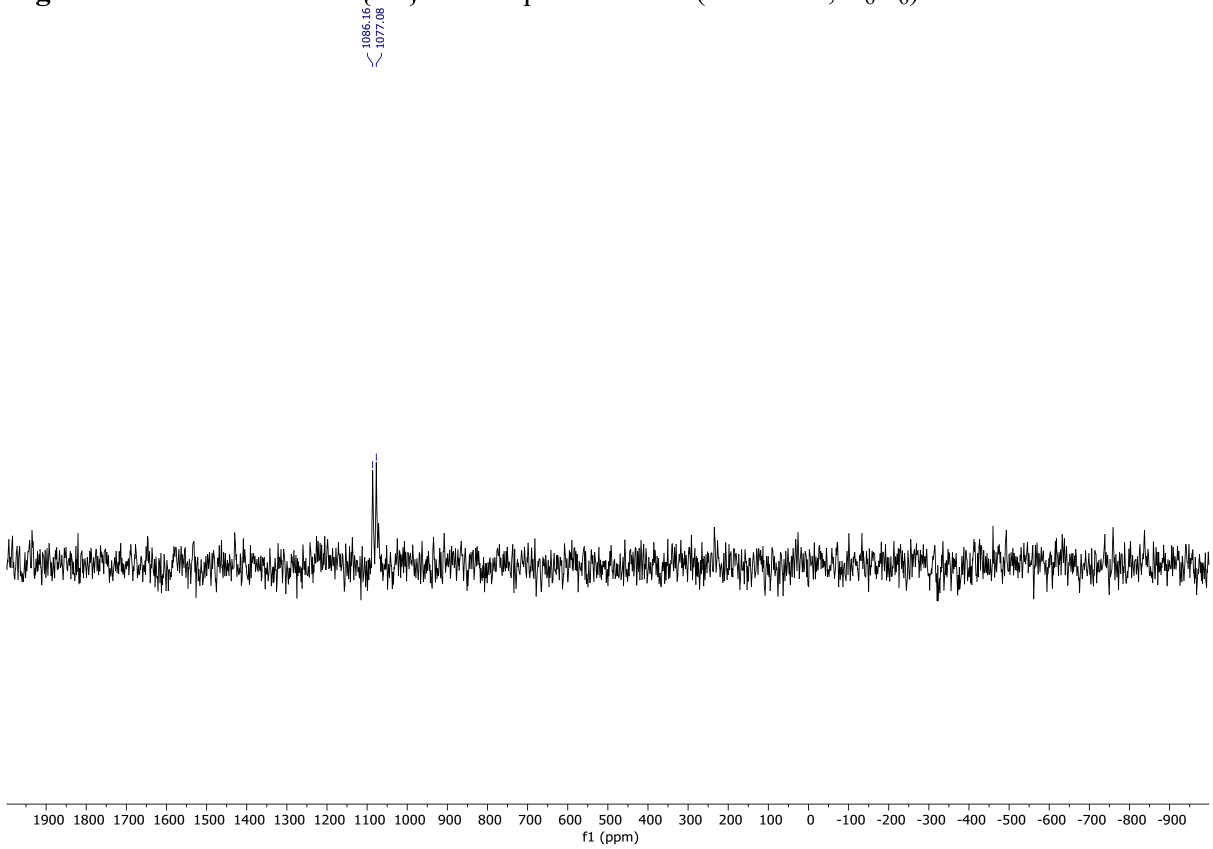

Figure S22. ^{77}Se NMR spectrum of **4** (95.4 MHz, d_8-tol, –45 °C).

3. High Resolution Mass Spectrum

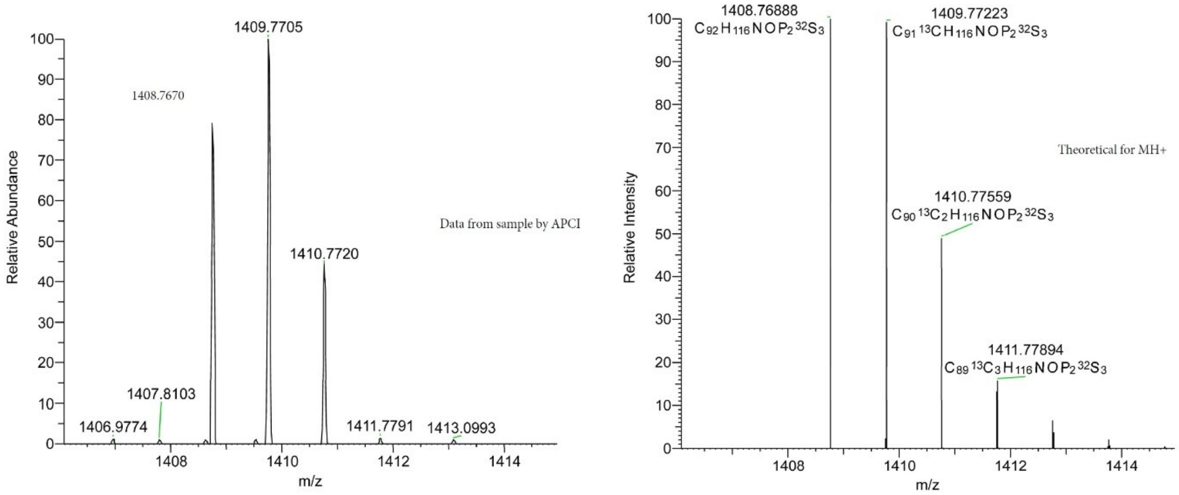

Figure S23. High resolution mass spectrum of **1** (left: experimental; right: calculated).

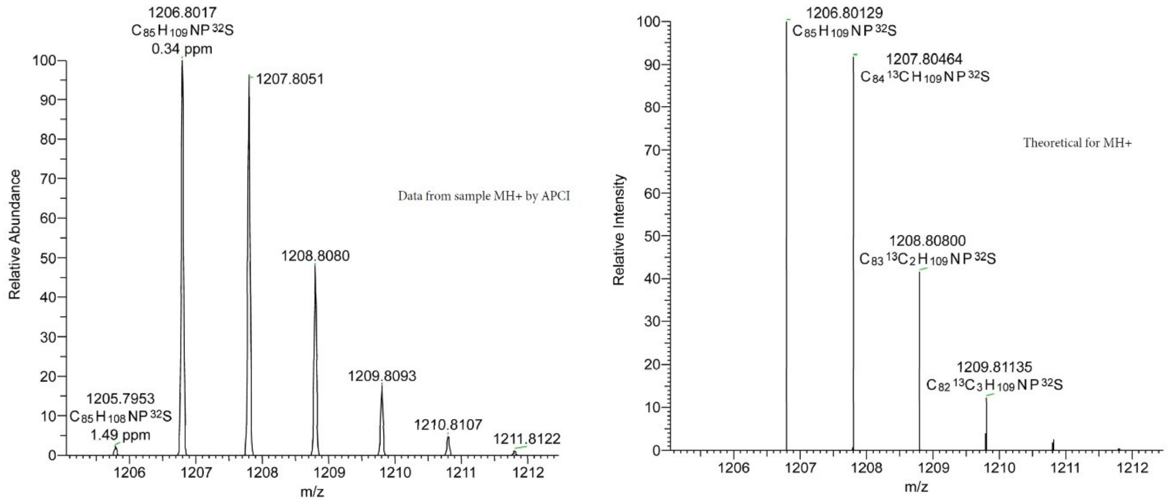

Figure S24. High resolution mass spectrum of **2** (left: experimental; right: calculated).

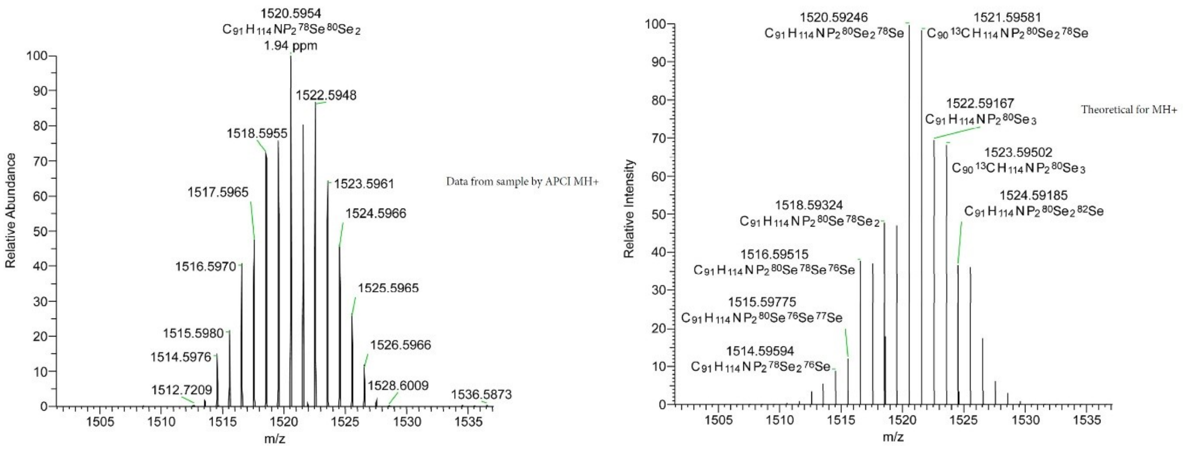

Figure S25. High resolution mass spectrum of **3** (left: experimental; right: calculated).

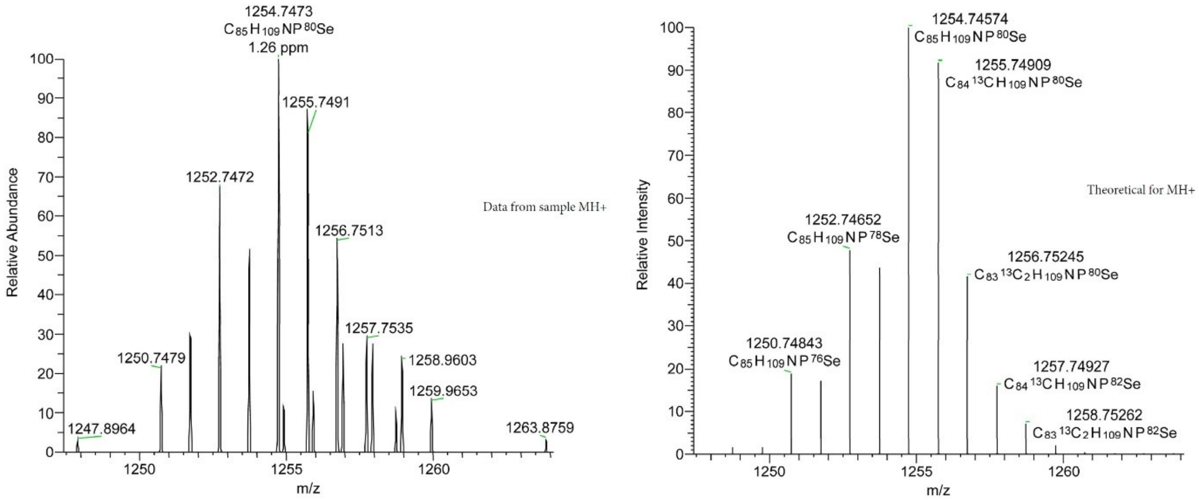

Figure S26. High resolution mass spectrum of **4** (left: experimental; right: calculated).

4. X-ray Crystallographic Data

4.1. Crystal structure data for **1**·hex (CCDC: 2444805)

A colorless, block-shaped specimen of **1**·hex (IUMSC 24344_sq) of approximate dimensions 0.175×0.192×0.332 mm^3, was placed on a Kapton mount with inert oil for crystal structure determination. The X-ray intensity data were measured with a microfocus sealed tube (λ = 1.54178 Å), a multilayer mirror monochromator, and a PhotonIII detector. The experiment was performed at 173(2) K using an Oxford Cryostream open-nitrogen-flow temperature control unit.

Data collection. The data collection was performed using 1° ω and φ scans, frame times between 80 and 1 s, and a detector distance of 40 mm. Overall, 2597 frames were collected with a total exposure time of 29.81 hours. The frames were integrated with the SAINT V8.41 package using a narrow-frame algorithm.[2] The integration of the data using a trigonal unit cell yielded 129694 reflections to a maximum θ angle of 68.47° (0.83 Å resolution), of which 18214 were independent (average redundancy 7.12, completeness = 99.9%, R_{int} = 8.77%, R_{sig} = 5.94%) and 13865 (76.1%) were greater than $2\sigma(F^2)$. The final cell constants of a = 62.7958(16) Å, b = 62.7958(16) Å, c = 13.0744(5) Å, α = 90°, β = 90°, γ = 120°, volume = 44649(3) $Å^3$, are based upon the refinement of the XYZ-centroids of 9753 reflections above 20 $\sigma(I)$ with 2.81° < 2θ < 68.28°. Data were corrected for absorption effects using the Multi-Scan method in SADABS 2016/2. The calculated minimum and maximum transmission coefficients (based on crystal size) are 0.674 and 0.806.[3] Additional crystal and refinement information can be found in the tables.

Structure solution and refinement. The space group $R\bar{3}$:H (148) was determined based on intensity statistics and systematic absences. The structure was solved by XT,

VERSION 2018/2 and refined with full-matrix least squares / difference Fourier cycles using SHELXL-2019/2; $Z = 18$ for the formula unit $C_{98}H_{129}NOP_2S_3$.[4,5] Non-hydrogen atoms were refined with anisotropic displacement parameters. The hydrogen atoms were placed in ideal positions and refined as riding atoms with relative isotropic displacement parameters.

Difference electron density indicated that partial and disordered solvent (hexane) was present in the structure. However, solvent models with strong sets of restraints and constraints did not converge to a chemically sensible structure. Therefore, the structure was investigated for solvent accessible areas.[6] Three large voids (symmetry equivalent) were found with a volume of 1939 Å^3 each (or 13% of the unit cell), containing in total 1131 electrons. For comparison, hexane occupies ca. 163 Å^3 with 50 electrons.[7] The contribution of the unidentified solvent to the structure factors was assessed by back-Fourier transformation[6] and the data were corrected accordingly. The refinement using the modified dataset improved the overall structure and R_1 by about 4%.

The final anisotropic full-matrix least-squares refinement on F^2 with 1109 variables against 18214 data points and 2058 restraints converged at $R_1 = 16.85\%$, for the observed data and $wR_2 = 48.92\%$ for all data. The goodness-of-fit on F^2 was 1.033. The largest peak in the final difference electron density synthesis was 2.24 e$^-$/Å^3 and the deepest hole was −0.77 e$^-$/Å^3 with an RMS deviation of 0.099 e$^-$/Å^3. On the basis of the final model, the calculated density was 1.001 g/cm^3 and F(000), 14580 e$^-$.

Table S1. Crystal data and structure refinement for **1**·hex **(CCDC: 2444805)**.

Empirical formula	$C_{98}H_{129}NOP_2S_3$	
Formula weight	1495.13	
Crystal color, shape, size	colorless block, 0.332 × 0.192 × 0.175 mm^3	
Temperature	173(2) K	
Wavelength	1.54178 Å	
Crystal system, space group	Trigonal, *R*–3	
Unit cell dimensions	*a* = 62.7958(16) Å	α = 90°
	b = 62.7958(16) Å	β = 90°
	c = 13.0744(5) Å	γ = 120°
Volume	44649(3) Å^3	
Z	18	
Density (calculated)	1.001 mg/m^3	
Absorption coefficient	1.292 mm^{-1}	
F(000)	14580	
**Data collection**		
Diffractometer	Venture D8, Bruker	
Source, Detector	Iµ3.0, Incoatec, Photon III	
Theta range for data collection	2.815 to 68.467°	
Index ranges	$-75 \le h \le 75$, $-75 \le k \le 75$, $-15 \le l \le 15$	
Reflections collected	129694	
Independent reflections	18214 [R_{int} = 0.0877]	
Observed Reflections	13865	
Completeness to theta = 67.679°	99.9 %	
**Solution and Refinement**		
Absorption correction	Semi-empirical from equivalents	
Max. and min. transmission	0.7531 and 0.5054	
Solution	Intrinsic methods	
Refinement method	Full-matrix least-squares on F^2	
Weighting scheme	$w = [\sigma^2 F_o^2 + AP^2 + BP]^{-1}$, with	
	$P = (F_o^2 + 2 F_c^2)/3$, A = 0.3155, B = 218.7800	
Data / restraints / parameters	18214 / 2057 / 1109	
Goodness-of-fit on F^2	1.033	
Final R indices [I > 2σ(I)]	R_1 = 0.1685, wR_2 = 0.4618	
R indices (all data)	R_1 = 0.1894, wR_2 = 0.4892	
Largest diff. peak and hole	2.237 and –0.773 e·Å^{-3}	

Goodness-of-fit = $[\sum[w(F_o^2 - F_c^2)^2]/(N_{observns} - N_{params})]^{1/2}$, all data.

$R_1 = \sum(|F_o| - |F_c|) / \sum |F_o|$. $wR_2 = [\sum[w(F_o^2 - F_c^2)^2] / \sum [w(F_o^2)^2]]^{1/2}$.

4.2. Crystal structure data for 2·hex (CCDC: 2444806)

A yellow, block-shaped specimen of **2**·hex (IUMSC 24342_sq) of approximate dimensions 0.342 × 0.154 × 0.042 mm^3 was placed onto the tip of a MiTeGen loop and mounted on a Bruker Venture D8 diffractometer equipped with a PhotonIII detector at 173(2) K.

Data collection. The data collection was carried out using Cu Kα radiation (multilayer mirror monochromator) with a frame time between 2 and 25 seconds and a detector distance of 40 mm. A collection strategy was calculated and complete data to a resolution of 0.83 Å (fifteen sets of frames) were collected with 1º ω and φ scans. A total of 3067 frames were collected. The total exposure time was 14.08 hours. The frames were integrated with the SAINT V8.40B package using a narrow-frame algorithm.[2] The integration of the data using a monoclinic unit cell yielded a total of 69240 reflections to a maximum θ angle of 68.61° (0.83 Å resolution), of which 14698 were independent (average redundancy 4.71, completeness = 99.8%, R_{int} = 9.29%, R_{sig} = 7.00%) and 13408 (91.2%) were greater than $2\sigma(F^2)$. The final cell constants of a = 15.2779(7) Å, b = 15.5539(8) Å, c = 16.9138(8) Å, α = 90°, β = 92.609(3)°, γ = 90°, volume = 4015.1(3) Å^3, are based upon the refinement of the XYZ-centroids of 9850 reflections above 20 σ(I) with 2.62° < 2θ < 68.41°. Data were corrected for absorption effects using the Multi-Scan method in SADABS 2016/2. The calculated minimum and maximum transmission coefficients (based on crystal size) are 0.757 and 0.965.[3] Table S2 contains additional crystal and refinement information.

Structure solution and refinement. The space group $P2_1$ (4) was determined based on intensity statistics and systematic absences. The structure was solved by XT, VERSION 2014/5 and refined with full-matrix least squares / difference Fourier cycles using SHELXL-2019/2; Z = 2 for the formula unit $C_{91}H_{122}NPS$.[4,5] Non-hydrogen atoms were refined with anisotropic

displacement parameters. The hydrogen atoms were placed in ideal positions and refined as riding atoms with relative isotropic displacement parameters. Remaining electron density indicated that additional partial solvent (hexane) was present in the structure. However, solvent models with strong sets of restraints and constraints did not converge to a chemically sensible structure. Therefore, the structure was investigated for solvent accessible areas.[6] Two voids were found in the unit cell (470 Å^3) to contain 98 electrons. For comparison, hexane occupies ca. 163 Å^3 with 50 electrons.[7] The contribution of the unidentified solvent to the structure actors was assessed by back-Fourier transformation[6] and the data were corrected accordingly. The refinement using the modified dataset improved the overall structure and R_{values}. The final anisotropic full-matrix least-squares refinement on F^2 with 871 variables against 14698 data points and 976 restraints converged at R_1 = 5.25%, for the observed data and wR_2 = 14.39% for all data. The goodness-of-fit on F^2 was 1.05. The largest peak in the final difference electron density synthesis was 0.377 e$^-$/Å^3 and the deepest hole was −0.249 e$^-$/Å^3 with an RMS deviation of 0.046 e$^-$/Å^3. On the basis of the final model, the calculated density was 1.069 g/cm^3 and F(000), 1412 e$^-$. The structure was refined as a 2-component inversion twin.

Table S2. Crystal data and structure refinement for **2**·hex **(CCDC: 2444806)**.

Empirical formula	$C_{91}H_{122}NPS$	
Formula weight	1292.92	
Crystal color, shape, size	yellow, 0.342 × 0.154 × 0.042 mm^3	
Temperature	173(2) K	
Wavelength	1.54178 Å	
Crystal system, space group	Monoclinic, $P2_1$	
Unit cell dimensions	a = 15.2779(7) Å	α = 90°
	b = 15.5539(8) Å	β = 92.609(3)°
	c = 16.9138(8) Å	γ = 90°
Volume	4015.1(3) $Å^3$	
Z	2	
Density (calculated)	1.069 mg/m^3	
Absorption coefficient	0.861 mm^{-1}	
$F(000)$	1412	
**Data collection**		
Diffractometer	Venture D8, Bruker	
Source, Detector	Iµ3.0, Incoatec, Photon III	
Theta range for data collection	2.895 to 68.605°	
Index ranges	$-16 \leq h \leq 18$, $-18 \leq k \leq 18$, $-20 \leq l \leq 20$	
Reflections collected	69240	
Independent reflections	14698 [R_{int} = 0.0929]	
Observed Reflections	13408	
Completeness to theta = 67.679°	99.8 %	
**Solution and Refinement**		
Absorption correction	Semi-empirical from equivalents	
Max. and min. transmission	0.7531 and 0.4850	
Solution	Intrinsic methods	
Refinement method	Full-matrix least-squares on F^2	
Weighting scheme	$w = [\sigma^2 F_o^2 + AP^2 + BP]^{-1}$, with	
	$P = (F_o^2 + 2 F_c^2)/3$, A = 0.0836, B = 0.4369	
Data / restraints / parameters	14698 / 976 / 871	
Goodness-of-fit on F^2	1.050	
Final R indices [I > 2σ(I)]	R_1 = 0.0525, wR_2 = 0.1387	
R indices (all data)	R_1 = 0.0573, wR_2 = 0.1439	
Twin domain ratio (inversion twin)	0.89(2) : 0.11(2)	
Largest diff. peak and hole	0.377 and –0.249 e·$Å^{-3}$	

Goodness-of-fit = $[\sum[w(F_o^2 - F_c^2)^2]/N_{observns} - N_{params})]^{1/2}$, all data.

$R_1 = \sum(|F_o| - |F_c|) / \sum |F_o|$. $wR_2 = [\sum[w(F_o^2 - F_c^2)^2] / \sum [w(F_o^2)^2]]^{1/2}$.

4.3. Crystal structure data for 3·hex (CCDC: 2444807)

A yellow, block-shaped specimen of **3**·hex (IUMSC 24338_sq) of approximate dimensions 0.154 × 0.234 × 0.405 mm^3, was placed on a Kapton mount with inert oil for crystal structure determination. The X-ray intensity data were measured on a Bruker Venture D8 Kappa diffractometer equipped with a microfocus sealed tube (λ = 1.54178 Å), a multilayer mirror monochromator, and a PhotonIII detector. The experiment was performed at 173(2) K using an Oxford Cryostream open-nitrogen-flow temperature control unit.

Data collection. The data collection was performed using 1° ω and φ scans, frame times between 2 and 50 s, and a detector distance of 40 mm. Overall, 2927 frames were collected with a total exposure time of 27.00 hours. The frames were integrated with the SAINT V8.40B package using a narrow-frame algorithm.[2] The integration of the data using a trigonal unit cell yielded 299590 reflections to a maximum θ angle of 68.78° (0.83 Å resolution), of which 18088 were independent (average redundancy 16.56, completeness = 100.0%, R_{int} = 27.80%, R_{sig} = 8.43%) and 10562 (58.4%) were greater than 2σ(F^2). The final cell constants of a = 62.514(3) Å, b = 62.514(3) Å, c = 13.0812(8) Å, α = 90°, β = 90°, γ = 120°, volume = 44272(5) Å^3, are based upon the refinement of the XYZ-centroids of 9899 reflections above 20 σ(I) with 2.45° < 2θ < 67.49°. Data were corrected for absorption effects using the Multi-Scan method in SADABS 2016/2. The calculated minimum and maximum transmission coefficients (based on crystal size) are 0.504 and 0.752.[3] Additional crystal and refinement information can be found in the tables.

Structure solution and refinement. The space group R–3:H (148) was determined based on intensity statistics and systematic absences. The structure was solved by SHELXT 2018/2 and refined with full-matrix least squares / difference Fourier cycles using SHELXL-2019/2; Z =

18 for the formula unit $C_{97}H_{127}NP_2Se_3$.[4,5] Non-hydrogen atoms were refined with anisotropic displacement parameters. The hydrogen atoms were placed in ideal positions and refined as riding atoms with relative isotropic displacement parameters.

Difference electron density indicated that partial and disordered solvent (hexane) was present in the structure. However, solvent models with strong sets of restraints and constraints did not converge to a chemically sensible structure. Therefore, the structure was investigated for solvent accessible areas.[6] Three large voids (symmetry equivalent) were found with a volume of 2553 $Å^3$ each (or 18% of the unit cell), containing in total 968 electrons. For comparison, hexane occupies ca. 163 $Å^3$ with 50 electrons.[7] The contribution of the unidentified solvent to the structure factors was assessed by back-Fourier transformation[6] and the data were corrected accordingly. The refinement using the modified dataset improved the overall structure and R_1 by about 3%.

The final anisotropic full-matrix least-squares refinement on F^2 with 1041 variables against 18088 data points and 1637 restraints converged at R_1 = 13.82%, for the observed data and wR_2 = 44.49% for all data. The goodness-of-fit on F^2 was 1.05. The largest peak in the final difference electron density synthesis was 2.20 $e^-/Å^3$ and the deepest hole was −0.79 $e^-/Å^3$ with an RMS deviation of 0.101 $e^-/Å^3$. On the basis of the final model, the calculated density was 1.084 g/cm^3 and $F(000)$, 15264 e^-. Disorder was refined for multiple parts of the structure using restraints and constraints.

Table S3. Crystal data and structure refinement for **3**·hex **(CCDC: 2444807)**.

Empirical formula	$C_{97}H_{127}NP_2Se_3$	
Formula weight	1605.81	
Crystal color, shape, size	yellow, 0.405 × 0.234 × 0.154 mm^3	
Temperature	173(2) K	
Wavelength	1.54178 Å	
Crystal system, space group	Trigonal, *R*–3	
Unit cell dimensions	*a* = 62.514(3) Å	α = 90°.
	b = 62.514(3) Å	β = 90°.
	c = 13.0812(8) Å	γ = 120°.
Volume	44272(5) $Å^3$	
Z	18	
Density (calculated)	1.026 mg/m^3	
Absorption coefficient	1.939 mm^{-1}	
F(000)	14364	
**Data collection**		
Diffractometer	Venture D8, Bruker	
Source, Detector	Iμ3.0, Incoatec, Photon III	
Theta range for data collection	2.448 to 68.780°.	
Index ranges	–75 ≤ h ≤ 73, –75 ≤ k ≤ 75, –15 ≤ l ≤ 15	
Reflections collected	299590	
Independent reflections	18088 [R_{int} = 0.2780]	
Observed Reflections	10562	
Completeness to theta = 67.679°	100.0 %	
**Solution and Refinement**		
Absorption correction	Semi-empirical from equivalents	
Max. and min. transmission	0.7531 and 0.4592	
Solution	Intrinsic methods	
Refinement method	Full-matrix least-squares on F^2	
Weighting scheme	w = $[\sigma^2 F_o^2 + AP^2 + BP]^{-1}$, with	
	P = $(F_o^2 + 2 F_c^2)/3$, A = 0.3259, B = 0.4000	
Data / restraints / parameters	18088 / 1637 / 1041	
Goodness-of-fit on F^2	1.051	
Final R indices [I > 2σ(I)]	R_1 = 0.1382, wR_2 = 0.4005	
R indices (all data)	R_1 = 0.1712, wR_2 = 0.4449	
Largest diff. peak and hole	2.201 and –0.794 e·$Å^{-3}$	

Goodness-of-fit = $[\sum[w(F_o^2 - F_c^2)^2]/N_{observns} - N_{params})]^{1/2}$, all data.

$R_1 = \sum(|F_o| - |F_c|) / \sum |F_o|$. $wR_2 = [\sum[w(F_o^2 - F_c^2)^2] / \sum [w(F_o^2)^2]]^{1/2}$.

4.4. Crystal structure data for **4**·hex (CCDC: 2444808)

A light red crystal of **4**·hex (IUMSC 24343_sq) of approximate dimensions 0.264 × 0.251 × 0.148 mm^3 was placed onto the tip of a MiTeGen loop and mounted on a Bruker Venture D8 diffractometer equipped with a PhotonIII detector at 173(2) K.

Data collection. The data collection was carried out using Cu Kα radiation (multilayer mirror monochromator) with a frame time between 1 and 30 seconds and a detector distance of 40 mm. A collection strategy was calculated and complete data to a resolution of 0.83 Å (eleven sets of frames) were collected with 1° ω and φ scans. A total of 3067 frames were collected. The total exposure time was 14.08 hours. The frames were integrated with the SAINT V8.40B package using a narrow-frame algorithm.[2] The integration of the data using a monoclinic unit cell yielded a total of 69240 reflections to a maximum θ angle of 68.61° (0.83 Å resolution), of which 14698 were independent (average redundancy 4.71, completeness = 99.8%, R_{int} = 9.29%, R_{sig} = 7.00%) and 13408 (91.2%) were greater than $2\sigma(F^2)$. The final cell constants of a = 15.2779(7) Å, b = 15.5539(8) Å, c = 16.9138(8) Å, α = 90°, β = 92.609(3)°, γ = 90°, volume = 4015.1(3) $Å^3$, are based upon the refinement of the XYZ-centroids of 9850 reflections above 20 σ(I) with 2.62° < 2θ < 68.41°. Data were corrected for absorption effects using the Multi-Scan method in SADABS 2016/2. The calculated minimum and maximum transmission coefficients (based on crystal size) are 0.754 and 0.850.[3] Table S4 contains additional crystal and refinement information.

Structure solution and refinement. The space group $P2_1$ (4) was determined based on intensity statistics and systematic absences. The structure was solved by XT, VERSION 2014/5 and refined with full-matrix least squares / difference Fourier cycles using SHELXL-2019/2; Z = 2 for the formula unit $C_{91}H_{122}NPSe$.[4,5] Non-hydrogen atoms were refined with anisotropic

displacement parameters. The hydrogen atoms were placed in ideal positions and refined as riding atoms with relative isotropic displacement parameters. Remaining electron density indicated that additional partial solvent (hexane) was present in the structure. However, solvent models with strong sets of restraints and constraints did not converge to a chemically sensible structure. Therefore, the structure was investigated for solvent accessible areas.[6] Two voids were found in the unit cell (~460 Å^3) to contain 100 electrons. For comparison, hexane occupies ca. 163 Å^3 with 50 electrons.[7] The contribution of the unidentified solvent to the structure factors was assessed by back-Fourier transformation[6] and the data were corrected accordingly. The refinement using the modified dataset improved the overall structure and R values. The final anisotropic full-matrix least-squares refinement on F^2 with 876 variables against 14027 data points and 1009 restraints converged at R_1 = 6.96%, for the observed data and wR_2 = 19.26% for all data. The goodness-of-fit on F^2 was 1.03. The largest peak in the final difference electron density synthesis was 0.889 e$^-$/Å^3 and the deepest hole was −1.110 e$^-$/Å^3 with an RMS deviation of 0.082 e$^-$/Å^3. On the basis of the final model, the calculated density was 1.106 g/cm^3 and F(000), 1448 e$^-$. The structure was refined as a 2-component inversion twin. This compound is isostructural to the S-containing analogue **2**.

Table S4. Crystal data and structure refinement for **4**·hex **(CCDC: 2444808)**.

Empirical formula	$C_{91}H_{122}NPSe$	
Formula weight	1339.82	
Crystal color, shape, size	light red plate, 0.264 × 0.251 × 0.148 mm^3	
Temperature	173(2) K	
Wavelength	1.54178 Å	
Crystal system, space group	Monoclinic, $P2_1$	
Unit cell dimensions	a = 15.2938(5) Å	α = 90°
	b = 15.5419(6) Å	β = 92.737(2)°
	c = 16.9381(7) Å	γ = 90°
Volume	4021.5(3) $Å^3$	
Z	2	
Density (calculated)	1.106 mg/m^3	
Absorption coefficient	1.135 mm^{-1}	
F(000)	1448	
**Data collection**		
Diffractometer	Venture D8, Bruker	
Source, Detector	Iμ3.0, Incoatec, Photon III	
Theta range for data collection	3.862 to 68.366°	
Index ranges	$-18 \le h \le 18$, $-18 \le k \le 18$, $-20 \le l \le 19$	
Reflections collected	49746	
Independent reflections	14027 [R_{int} = 0.0937]	
Observed Reflections	12317	
Completeness to theta = 67.679°	99.7 %	
**Solution and Refinement**		
Absorption correction	Semi-empirical from equivalents	
Max. and min. transmission	0.7531 and 0.4924	
Solution	Intrinsic methods	
Refinement method	Full-matrix least-squares on F^2	
Weighting scheme	$w = [\sigma^2 F_o^2 + AP^2]^{-1}$, with	
	$P = (F_o^2 + 2 F_c^2)/3$, A = 0.1425	
Data / restraints / parameters	14027 / 1009 / 876	
Goodness-of-fit on F^2	1.033	
Final R indices [I > 2σ(I)]	R_1 = 0.0696, wR_2 = 0.1836	
R indices (all data)	R_1 = 0.0770, wR_2 = 0.1926	
Twin domain ratio (inversion twin)	0.88(2) : 0.12(2)	
Largest diff. peak and hole	0.889 and –1.110 e·$Å^{-3}$	

Goodness-of-fit = $[\sum[w(F_o^2 - F_c^2)^2]/N_{observns} - N_{params})]^{1/2}$, all data.

$R_1 = \sum(|F_o| - |F_c|) / \sum |F_o|$. $wR_2 = [\sum[w(F_o^2 - F_c^2)^2] / \sum [w(F_o^2)^2]]^{1/2}$.

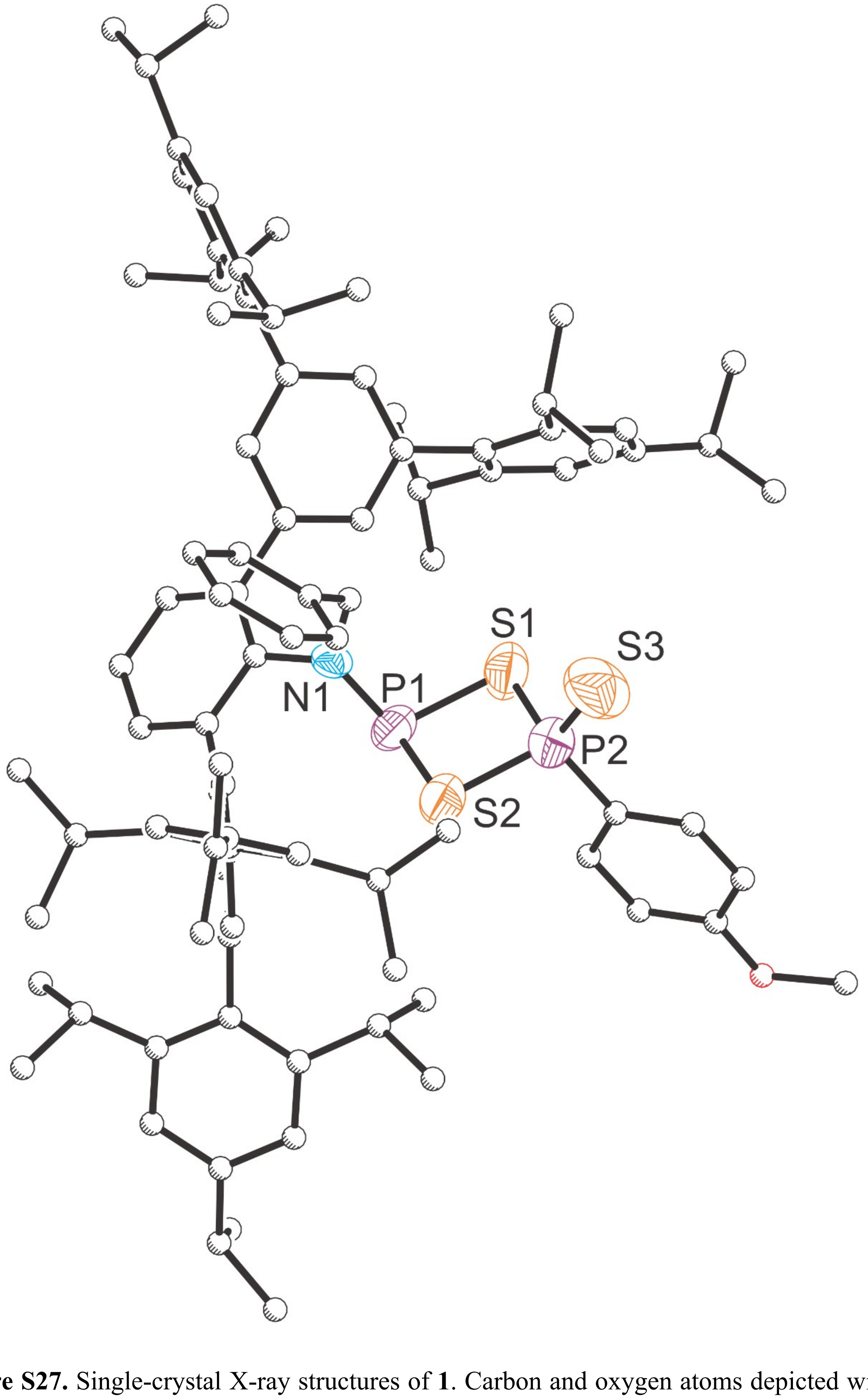

Figure S27. Single-crystal X-ray structures of **1**. Carbon and oxygen atoms depicted with an arbitrary radius. Thermal ellipsoids pictured at 30% probability level. Positional disorder and hydrogen atoms removed for clarity.

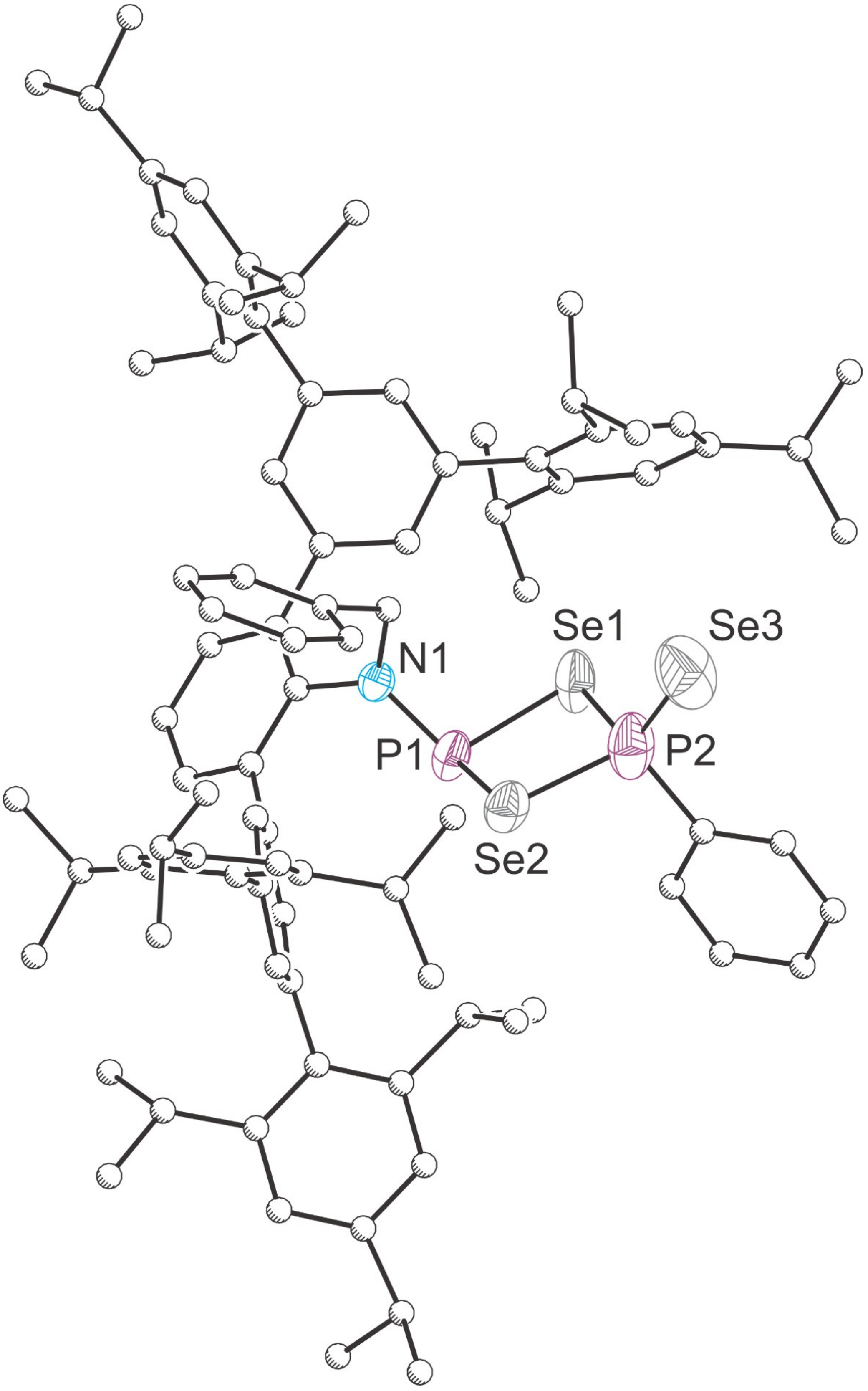

Figure S28. Single-crystal X-ray structures of **3**. Carbon atoms depicted with an arbitrary radius. Thermal ellipsoids pictured at 30% probability level. Positional disorder and hydrogen atoms removed for clarity.

5. Computational Details

Geometry optimizations were carried out using the Gaussian 16 package with the M06-2X functional.[8, 9] The def2-SVP basis set was used for all the atoms. [10,11] Frequency calculations at the same level of theory were performed to identify the number of imaginary frequencies (zero for local minimum and one for transition states), and provide the thermal corrections of Gibbs free energy. Single-point energy calculations were performed at the M06-2X/def2-TZVP level of theory for the modelling of compounds in solution (benzene). The gas-phase geometry was used for all the solution phase calculations. The SMD method was used with the corresponding solvent, while Bondi radii were chosen as the atomic radii to define the molecular cavity.[12,13] The corrections of Gibbs free energy from frequency calculations were added to the single-point energies to obtain the Gibbs free energy in solution. All the energies reported in the paper correspond to the reference state of 1 mol/L, 298K. Natural bond orbital (NBO) calculations were carried out using NBO 7.0 program at the M06-2X/def2-SVP level of theory.[14] Optimized structures and orbitals were visualized using Chemcraft.[15]

5.1 Energy Calculation for *cis-trans* isomerization and thermodynamical profile of equilibrium between 2, 3 and LR

Table S5. Energies of Intermediates and Transition States.

Species	Thermal Corrections of Gibbs Free Energies (Hartree)	Solvation Energies (Hartree)
**2-*trans***	1.585163	-4098.297182
**ts–2**	1.584057	-4098.263047
**2-*cis***	1.586667	-4098.297751
**4-*trans***	1.584158	-6101.688553
**ts-4**	1.582865	-6101.652436
**4-*cis***	1.583004	-6101.687109
**1**	1.703693	-5582.270251
**LR**	0.207821	-2967.914383
**DMAP**	0.12982	-382.237078
**$ArPS_2$(DMAP)**	0.245497	-1866.213049

5.2 NBO orbital for 2 and 4

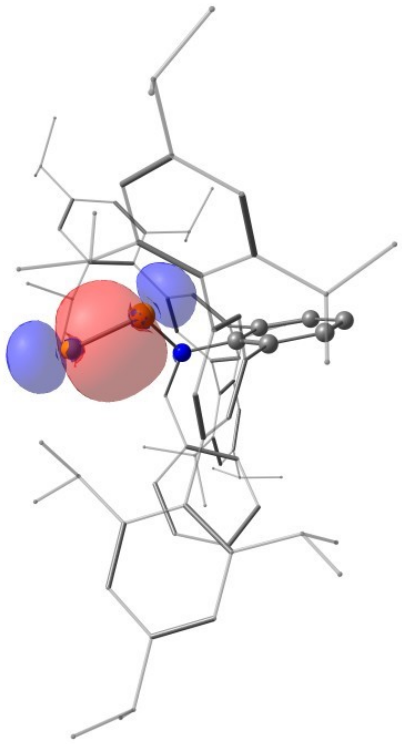

σ-orbital in **2** (NBO 274)

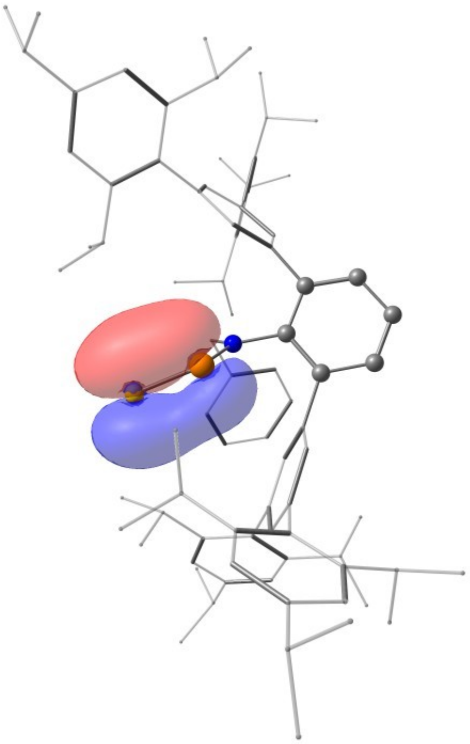

π-orbital in **2** (NBO 275)

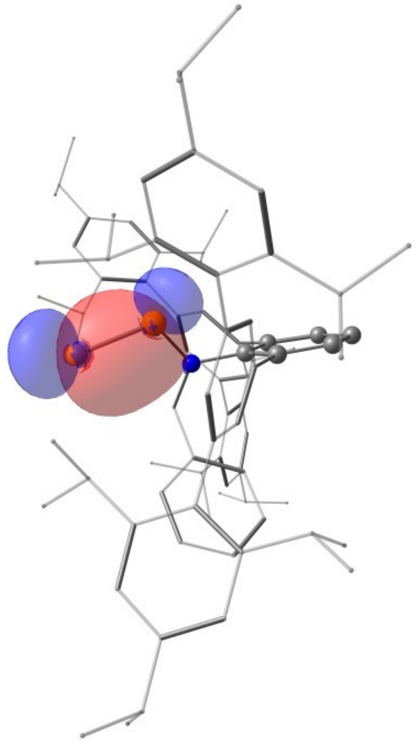

σ-orbital in **4** (NBO 283)

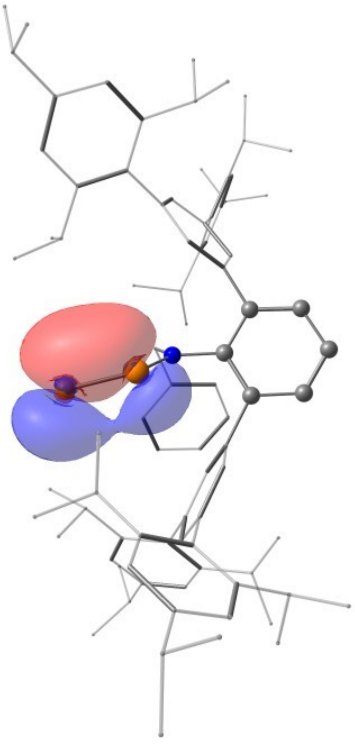

π-orbital in **4** (NBO 284)

Figure S29. σ- and π-NBO orbital of **2** and **4**.

5.3 Energy profiles for the equilibrium between 1,2 and LR

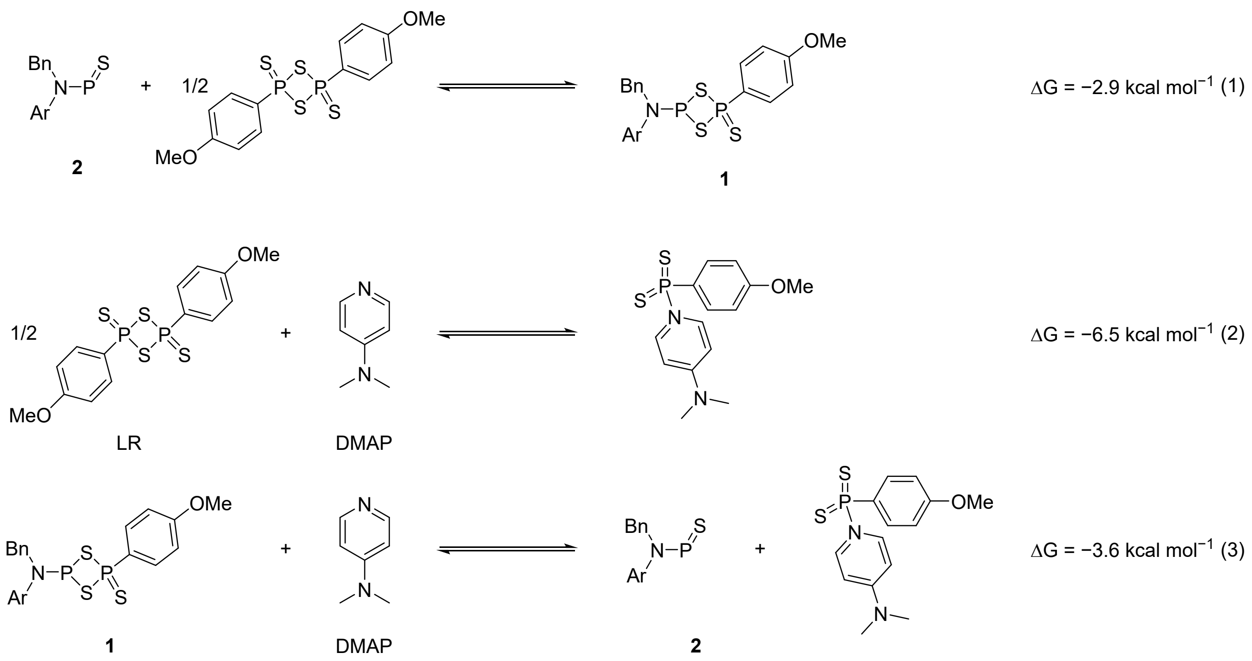

Scheme S8. DFT calculated energy profiles for the equilibrium between **1**, **2** and LR.

6. References

1 C. Hu, N. H. Rees, M. Pink and J. M. Goicoechea, *Nat. Chem.*, 2024, **16**, 1855–1860.

2 SAINT V8.41 (2024), Bruker AXS, Madison, WI, USA.

3 L. Krause, R. Herbst-Irmer, G. M. Sheldrick, and D. Stalke, *J. Appl. Cryst.*, 2015, **48**, 3–10.

4 G. M. Sheldrick, *Acta Cryst.*, 2015, **A71**, 3–8.

5 G. M. Sheldrick, *Acta Cryst.*, 2015, **C71**, 3–8.

6 A. L. Spek, *Acta Cryst.*, 2015, **C71**, 9–18.

7 A. Immirzi and B. Perini, *Acta Cryst.*, 1977, **A33**, 216–218.

8 Gaussian 16, Revision C.01, M. J. Frisch, G. W. Trucks, H. B. Schlegel, G. E. Scuseria, M. A. Robb, J. R. Cheeseman, G. Scalmani, V. Barone, G. A. Petersson, H. Nakatsuji, X. Li, M. Caricato, A. V. Marenich, J. Bloino, B. G. Janesko, R. Gomperts, B. Mennucci, H. P. Hratchian, J. V. Ortiz, A. F. Izmaylov, J. L. Sonnenberg, D. Williams-Young, F. Ding, F. Lipparini, F. Egidi, J. Goings, B. Peng, A. Petrone, T. Henderson, D. Ranasinghe, V. G. Zakrzewski, J. Gao, N. Rega, G. Zheng, W. Liang, M. Hada, M. Ehara, K. Toyota, R. Fukuda, J. Hasegawa, M. Ishida, T. Nakajima, Y. Honda, O. Kitao, H. Nakai, T. Vreven, K. Throssell, J. A. Montgomery, Jr., J. E. Peralta, F. Ogliaro, M. J. Bearpark, J. J. Heyd, E. N. Brothers, K. N. Kudin, V. N. Staroverov, T. A. Keith, R. Kobayashi, J. Normand, K. Raghavachari, A. P. Rendell, J. C. Burant, S. S. Iyengar, J. Tomasi, M. Cossi, J. M. Millam, M. Klene, C. Adamo, R. Cammi, J. W. Ochterski, R. L. Martin, K. Morokuma, O. Farkas, J. B. Foresman and D. J. Fox, Gaussian, Inc., Wallingford CT, 2016.

9 Y. Zhao and D. G. Truhlar, *Theor. Chem. Acc.*, 2008, **120**, 215–241.

10 F. Weigend and R. Ahlrichs, *Phys. Chem. Chem. Phys.*, 2005, **7**, 3297–3305.

11 F. Weigend, *Phys. Chem. Chem. Phys.*, 2006, **8**, 1057–1065.

12 A. V. Marenich, C. J. Cramer and D. G. Truhlar, *J. Phys. Chem. B*, 2009, **113**, 6378–6396.

13 A. Bondi, *J. Phys. Chem.*, 1964, **68**, 441–451.

14 NBO 7.0. E. D. Glendening, J. K. Badenhoop, A. E. Reed, J. E. Carpenter, J. A. Bohmann, C. M. Morales, P. Karafiloglou, C. R. Landis and F. Weinhold, University of Wisconsin, Madison WI, 2018.

15 ChemCraft - Graphical Software for Visualization of Quantum Chemistry Computations, Version 1.8. http://www.chemcraftprog.com.
